# Conserved CD8 T cell vaccines without B cell epitopes drive robust protection against SARS-CoV-2 that is enhanced by intranasal boost

**DOI:** 10.1126/sciadv.adx0037

**Published:** 2025-11-21

**Authors:** Genghao Chen, Thao Nguyen, Lindsay G. A. McKay, Sravya Sowdamini Nakka, Pan Hu, Julia McBride, Anthony C. Liang, Rachel Olson, James J. Moon, Andrew D. Luster, Anthony Griffiths, Stephen J. Elledge

**Affiliations:** ^1^ Division of Genetics, Department of Medicine, Howard Hughes Medical Institute, Brigham and Women’s Hospital, Boston, MA 02115, USA.; ^2^ Department of Genetics, Harvard Medical School, Boston, MA 02115, USA.; ^3^ Center for Immunology and Inflammatory Diseases, Massachusetts General Hospital, Boston, MA 02129, USA.; ^4^ Division of Rheumatology, Allergy, and Immunology, Massachusetts General Hospital, Boston, MA 02129, USA.; ^5^ National Emerging Infectious Diseases Laboratories (NEIDL) and Department of Virology, Immunology, and Microbiology, Boston University Medical School, Boston, MA 02118, USA.; ^6^ Department of Veterinary Pathobiology, University of Missouri, Columbia, MO 65211, USA.; ^7^ Harvard Medical School, Boston, MA 02115, USA.; ^8^ Division of Pulmonary and Critical Care Medicine, Massachusetts General Hospital, Boston, MA 02129, USA.

## Abstract

The emergence of SARS-CoV-2 variants has challenged the current spike protein–focused COVID-19 vaccine strategy due to neutralizing antibody escape and waning antibody-mediated immunity. In contrast, T cell–mediated immunity targeting conserved epitopes may offer broad and long-lasting protection. However, whether T cells alone can provide sufficient protection remains unclear. Here, we identified both Omicron BA.1–specific and ancestral (Wuhan)–conserved CD8 T cell epitopes in the SARS-CoV-2 spike protein and evaluated them as carrier-protein fusion vaccines in mouse models. Subcutaneous immunizations with two CD8 epitope peptides substantially lowered lung viral load and conferred protection against low-dose viral challenge, but not against high-dose challenge. Notably, intranasal boosting—with or without adjuvant—enhanced lung resident memory T cell responses and conferred potent, durable protection against high-dose infection. These findings emphasize the importance of mucosal vaccination to boost protective T cell immunity against SARS-CoV-2 and support the potential of T cell–based vaccines targeting conserved epitopes for broad immunity against SARS-CoV-2 and other respiratory viral threats.

## Teaser

Conserved CD8 T cell vaccines provide robust protection against SARS-CoV-2 that is enhanced by intranasal boosting.

## INTRODUCTION

The emergence of the severe acute respiratory syndrome coronavirus 2 (SARS-CoV-2) in 2019 prompted an unprecedented vaccine development effort to mitigate the COVID-19 pandemic. Among the most successful outcomes of this effort were the mRNA–lipid nanoparticle (LNP) vaccines, which demonstrated more than 90% efficacy against symptomatic disease in initial clinical studies (*1*, *2*). These vaccines, encoding the SARS-CoV-2 spike protein, primarily aim to induce neutralizing antibodies that can prevent viral entry into host cells. Animal and clinical studies demonstrated the capacity of mRNA vaccines to elicit high levels of neutralizing antibody responses (*3*). The remarkable efficacy of spike mRNA vaccines toward initial SARS-CoV-2 variants highlighted the importance of neutralizing antibodies in driving protection. However, antibodies alone cannot fully explain protective immunity against SARS-CoV-2. Individuals with B cell deficiencies can recover from COVID-19, sometimes with mild symptoms (*4*, *5*), suggesting that antibody-independent immunity can effectively control SARS-CoV-2 infection. This is likely due in part to contributions from cellular immunity generated by CD4 and CD8 T cells. Growing evidence supports the critical role of CD8 T cells in the control and clearance of SARS-CoV-2 infections, with early onset of SARS-CoV-2–specific CD8 T cell responses being the best correlate of low disease severity (*6*, *7*). Furthermore, depletion of CD8 T cells in nonhuman primates partially abrogated protection provided by prior infection or vaccination (*8*, *9*). Mechanistically, CD8 T cells could recognize and kill infected cells that present viral peptides on major histocompatibility complex class I (MHC-I), thereby halting viral replication and intrahost dissemination. Vaccines that induce a strong CD8 T cell response might elicit sufficient protection by limiting disease severity and transmission, even in the absence of humoral immunity.

The emergence of highly transmissible variants, such as Omicron, that evade neutralizing antibody responses has challenged the current antibody-centric vaccine design. Antibody-mediated protection is further compromised by waning titers, highlighting the need for complementary strategies that can provide durable and broad-spectrum protection. T cell immunity exhibits greater durability and resistance to viral escape compared to antibodies (*10*–*12*), with reports of antiviral T cell responses persisting for over a decade after SARS-CoV infection (*13*). Targeting conserved and mutationally constrained T cell epitopes, both within and outside of the spike protein, presents a promising approach for achieving broad and durable protection against current and future viral variants.

Several studies have explored SARS-CoV-2 T cell vaccines with diverse designs and varying efficacies in animal models (*14*–*16*). BioNTech developed a T cell–directed, nonspike vaccine BNT162b4 which is an mRNA encoding viral protein segment predicted to be rich in T cell epitopes across multiple MHC-I haplotypes (*16*). However, this strategy induces both CD4 and CD8 T cell responses, making it difficult to pinpoint the specific contributions of each T cell subset to vaccine-induced protection. Pardieck *et al.* (*14*) reported protection from low-dose SARS-CoV-2 challenge after a third subcutaneous immunization with a synthetic long peptide (SLP) vaccine containing an immunodominant CD8 T cell epitope (Wuhan-1 S539-546). However, its efficacy against higher viral doses—where spike mRNA vaccines provide robust protection—was not assessed (*14*).

One major limitation of current intramuscularly (IM) administered vaccines is the limited ability to induce potent mucosal immunity compared to natural infection (*17*, *18*). Mucosal immunity in the respiratory tract is crucial for protection against SARS-CoV-2 and other respiratory viruses. It has potential to not only prevent and reduce disease but also limit transmission through decreasing virus replication and shedding in the respiratory mucosa (*19*, *20*). While intranasal (IN) and other respiratory tract vaccination routes hold promise for inducing mucosal immunity, its development is hindered by poor immunogenicity and tolerability (*20*, *21*). While adjuvants can improve vaccine immunogenicity and enable dose sparing, their IN administration can trigger excessive inflammation and adverse reactions. For example, enterotoxin-based adjuvants in IN influenza vaccines have been associated with cases of Bell’s palsy (*21*, *22*). This highlights the critical need for safe and effective adjuvants, or preferably adjuvant-free (AF) strategies, for IN vaccination. Although mRNA-LNP vaccines have shown remarkable immunogenicity via IM routes, their efficacy and tolerability following IN administration require thorough investigation. In one study, IN LNP delivery was found to be highly inflammatory and lethal at high doses (*23*). Therefore, protein-based vaccines remain a favorable option for IN administration due to good safety profiles, low cost of production, and ease of transportation. While recent preclinical studies demonstrated efficacy of IN spike protein boosters in protecting against SARS-CoV-2 (*24*–*26*), the relative contribution of T cells (relative to antibodies) to protective mucosal immunity remains unclear, and whether mucosal T cell response alone could offer sufficient protection still requires investigation.

To harness the potential of T cell and mucosal immunity for vaccine development, we first identified previously unknown CD8 T cell epitopes, including those containing Omicron BA.1 mutations, in mouse models. We then developed carrier-protein fusion vaccines designed to induce only T cell responses targeting conserved epitopes shared between BA.1 and the Wuhan strain. This allowed us to assess the protective efficacy of T cell immunity in the absence of preexisting antibodies. We further explored the impact of IN boosting, in the presence or absence of adjuvants, on mucosal T cell responses and protective efficacy against SARS-CoV-2. Our findings highlight the potential of T cell–based vaccines, especially when combined with mucosal delivery, to provide durable and broad-spectrum protection against SARS-CoV-2 and future emerging viral threats particularly in scenarios where viruses completely escape from antibody recognition.

## RESULTS

### Mapping CD8 T cell epitopes of Omicron BA.1 spike protein in C57BL/6 mice

**Fig. 1.**
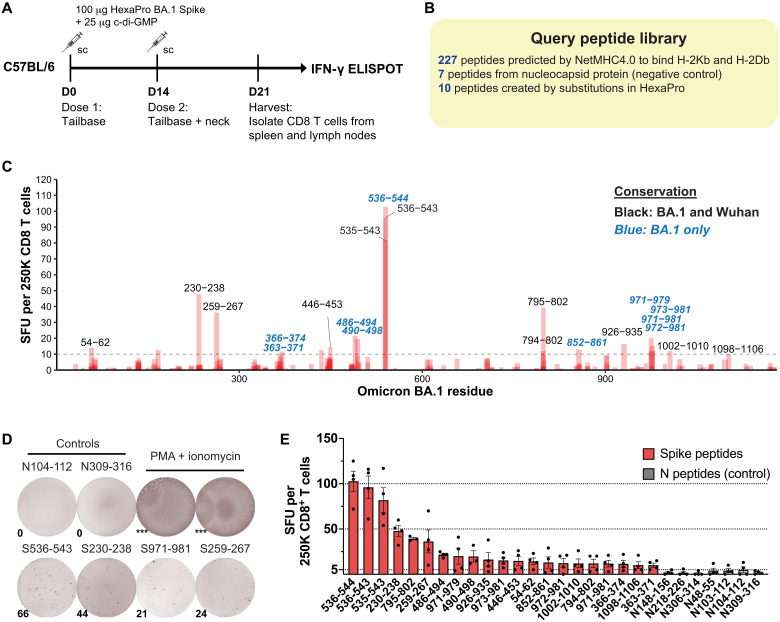
Mapping of BA.1 spike CD8 T cell epitopes in C57BL/6 mice. (**A**) Immunization scheme for epitope mapping of CD8 T cell epitopes in C57BL/6 mice. Male and female C57BL/6 mice (8 to 10 mice per replicate, four replicates) were immunized with 100 μg of HexaPro Omicron BA.1 spike protein with 25 μg of c-di-GMP adjuvant twice. The first dose was given at the tail base; the second was given at both the tail base and the back of the neck. On day 21 (D21), spleens and lymph nodes from mice were pooled, and CD8 T cells were isolated to perform IFN-γ ELISPOT. (**B**) Composition of the query peptide library used for epitope mapping. (**C**) ELISPOT results. *Y* axis shows spot-forming units (SFU) per 250,000 CD8 T cells, averaged from four independent replicates. Peptides that contain mutations from Wuhan-1 are highlighted in blue and italic. (**D**) Representative ELISPOT images for selected peptides. *** denotes uncountable (significantly higher spot counts). PMA, phorbol 12-myristate 13-acetate. (**E**) Quantification of ELISPOT results. Red, spike peptides with a response above background; gray, control peptides from N protein. Mean ± SEM. Line at 5 SFU denotes the background level. sc, subcutaneous.

To identify CD8 T cell epitopes, C57BL/6 mice were immunized twice with recombinant BA.1 spike protein combined with the cyclic diguanylate (c-di-GMP) adjuvant. CD8 T cells were isolated from spleen and lymph nodes, and reactivity to individual query peptides was assessed using interferon-γ (IFN-γ) ELISPOT (enzyme-linked immunoSPOT) (Fig. 1A). Nucleocapsid (N) peptides were used as negative controls since N was not included in the immunizations. The query peptide library consists of 244 query peptides: 227 peptides from Omicron BA.1 SARS-CoV-2 spike (S) protein, 7 peptides from N protein, and 10 peptides containing stabilizing proline mutations in the HexaPro spike antigen (Fig. 1B) (*27*). These peptides were selected on the basis of NetMHC4.0-predicted binding affinity to mouse MHC-I alleles, H-2K^b^ and H-2D^b^ (*28*, *29*).

The epitope mapping screen revealed 21 peptides with significant reactivity, yielding a total of 16 nonoverlapping putative epitopes (Table 1, *putative epitopes). Among these, nine epitopes are conserved between BA.1 and Wuhan, while seven epitopes are BA.1 specific, containing amino acid differences compared to Wuhan. Of the nine conserved epitopes, two were immunodominant epitopes that were previously reported by Zhuang *et al.* (*30*)—BA1.S259-267 (AAAYYVGYL, S262-270 in Wuhan) and BA1.S536-543 (VNFNFNGL, S539-S546 in Wuhan)—and seven epitopes were found in this study (Fig. 1C, black: epitopes shared between BA.1 and Wuhan, blue: BA.1-specific epitopes). All seven BA.1-specific epitopes have not been previously reported, including BA1.S486-494 (YFPLRSYSF) and BA1.S971-981 (SSVLNDIFSRL) (Fig. 1, C to E). To further validate these findings, we performed a cell-based surface MHC stabilization assay to confirm the binding and MHC restriction of the mapped epitope peptides (fig. S1, A and B, and table S1) (*31*). Our study identified previously unknown and conserved mouse CD8 T cell epitopes within the Omicron BA.1 spike protein. Together with our companion study on BA.1 CD4 T cell epitopes (*32*), this collection of T cell epitopes will enable tracking of both conserved and BA.1-specific T cell responses and facilitate future immunological studies in mouse models regarding variant-specific T cells.

**Table 1.** Summary of mapped CD8 T cell epitopes. Residue position is in reference to BA.1 spike protein.

Peptide	Sequence	Conserved with Wuhan?	Previously reported?
**S230-238** *	INITRFQTL	Yes	No
**S259-267** *	AAAYYVGYL	Yes	Yes
**S363-371** *	SVLYNLAPF	No	No
**S366-374** *	YNLAPFFTF	No	No
**S446-453** *	YNYLYRLF	Yes	No
**S486-494** *	YFPLRSYSF	No	No
**S490-498** *	RSYSFRPTY	No	No
**S535-543**	CVNFNFNGL	Yes	Yes
S536-543*	VNFNFNGL	Yes	Yes
**S536-544**	VNFNFNGLK	No	No
**S54-62** *	LFLPFFSNV	Yes	No
**S794-802**	FGGFNFSQI	Yes	No
**S795-802** *	GGFNFSQI	Yes	No
**S852-861** *	FKGLTVLPPL	No	No
**S926-935** *	SAIGKIQDSL	Yes	No
**S971-979** *	SSVLNDIFS	No	No
**S971-981** *	SSVLNDIFSRL	No	No
**S972-981**	SVLNDIFSRL	No	No
**S973-981**	VLNDIFSRL	No	No
**S1002-1010** *	QTYVTQQLI	Yes	No
**S1098-1106** *	HWFVTQRNF	Yes	No

* Putative epitopes.

### Vaccinations with TTR-EP fusion immunogens induce strong and polyfunctional CD8 and CD4 T cell responses

**Fig. 2.**
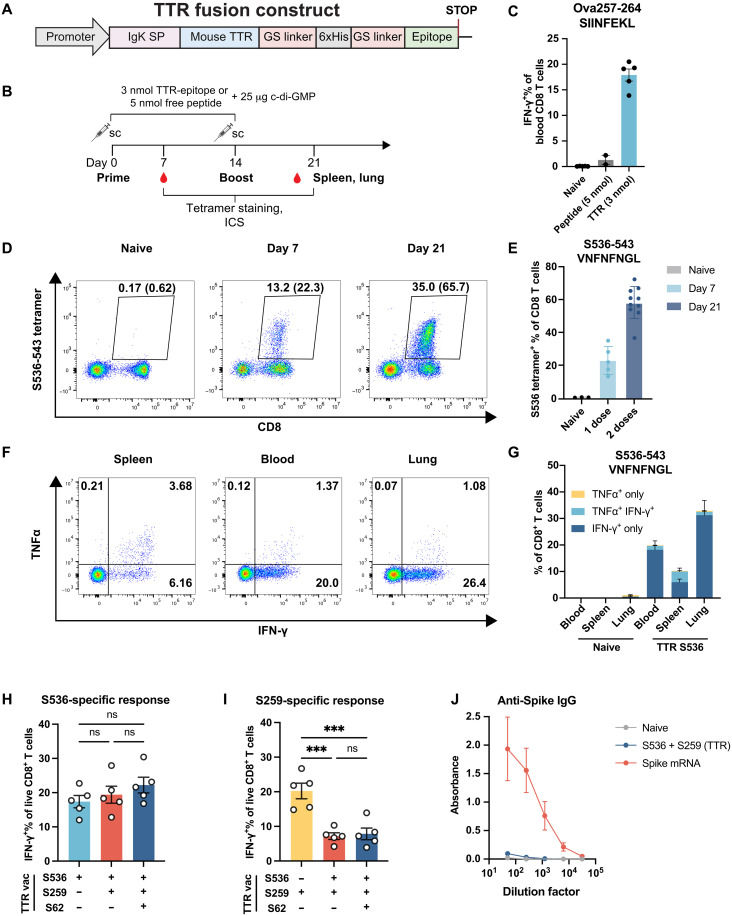
Vaccinations with TTR-EP fusion immunogens induce strong and polyfunctional CD8 and CD4 T cell responses. (**A**) Expression construct of TTR fusion proteins. IgK, immunoglobulin kappa. SP, signal peptide. GS linker, Glycine-serine linker (**B**) Immunization scheme for experiments in (C) to (J). Female C57BL/6 mice received immunizations on days 0 and 14. On day 7, peripheral blood was collected, and on day 21, blood, spleen, and lung were collected to evaluate T cell response via tetramer staining or ICS. (**C**) ICS of blood CD8 T cells following Ova257-264 immunizations either as free peptide or as TTR fusion. Mice were immunized with two doses of TTR Ova257-264 (3 nmol) or carrier-free Ova257-264 peptide (5 nmol). Blood was collected on day 21 and restimulated with Ova257-264 peptide. (**D** and **E**) Tetramer staining of blood CD8 T cells in naïve and TTR S536-543 immunized mice on day 7 and day 21. Gated on live CD3^+^ T cells. Percentage of CD8^+^S536-tetramer^+^ in CD3^+^ cells is shown. Percentage of S536-tetramer^+^ in CD3^+^CD8^+^ is shown in parentheses and quantified in (E) with mean ± SD. (**F** and **G**) ICS of spleen, blood, and lung CD8 T cells on day 21 following two doses of TTR S536 immunizations. Cytokine^+^ cells are quantified in (G). Mean ± SEM is shown. *N* = 5. (**H** and **I**) ICS of blood CD8 T cells after immunizations with single or combinations of epitopes. (H) IFN-γ^+^ CD8 T cell response to S536-543 peptide restimulation. (I) IFN-γ^+^ CD8 T cell response to S259-267 peptide restimulation. One-way analysis of variance (ANOVA) and Tukey’s test was used. (**J**) Serum anti-Spike IgG levels of vaccinated mice on day 21 measured by enzyme-linked immunosorbent assay. Mean ± SD of absorbance values at each serum dilution is shown. ns, not significant.

To generate T cell responses to spike epitopes in the absence of antibodies, we used a previously described carrier-protein fusion strategy in which epitope-containing peptides (EPs) are fused to the C terminus of mouse transthyretin (TTR), an abundant serum protein, to enhance immunogenicity through lymph node retention and favorable pharmacokinetics (Fig. 2A) (*33*). His-tagged TTR fusions were recombinantly expressed in mammalian cells and purified via affinity chromatography (fig. S2A). To confirm the immunogenicity of TTR-EP fusions, we immunized C57BL/6 mice subcutaneously with two doses of the immunodominant H-2K^b^–restricted ovalbumin epitope (Ova257-264, SIINFEKL) fused to TTR and assessed responses by intracellular cytokine staining (ICS) following ex vivo peptide restimulation (Fig. 2B). Notably, TTR fusion (3 nmol) significantly enhanced SIINFEKL-specific CD8 T cell responses (17.9% versus 1.2%) compared to peptide alone (5 nmol), confirming the immunogenicity-enhancing properties of TTR fusions (Fig. 2C). The immunogenicity of TTR fusion was not affected by sex, as both male and female mice immunized with TTR Ova257-264 exhibited similar levels of Ova257-264–specific CD8 T cell responses (fig. S2B).

Next, we examined the ability of TTR fusion to elicit CD8 T cell responses against SARS-CoV-2 epitopes. TTR S536-543 immunization led to 23.3 and 58.2% S536-tetramer^+^ CD8 T cells within total CD8 T cells in the blood on day 7 and day 21, respectively (Fig. 2, D and E). These S536-specific T cells predominantly exhibited an effector memory (T_EM_) phenotype (CD62L^−^CD44^+^) (fig. S2C). On day 21, S536-specific T cells [IFN-γ^+^ or tumor necrosis factor–α^+^ (TNFα^+^)] were detected in the blood, spleen, and lung at frequencies of 19.9, 10.3, and 32.9%, respectively (Fig. 2, F and G). Moreover, a large proportion of S536- and S259-specific T cells was CD107a^+^, suggesting that they were capable of degranulation upon antigen stimulation (fig. S2, E and F). Subcutaneous immunization with TTR BA1.S971-981 (SSVLNDIFSRL), a BA.1-specific epitope, generated splenic T cells that responded to S971-981 or S972-981 peptides in the ICS assay (fig. S2D). These findings demonstrate that subcutaneous immunization with TTR-EP fusions effectively induces polyfunctional SARS-CoV-2–specific CD8 T cell responses that circulate and, importantly, infiltrate the lung.

To assess CD4 T cell responses, we generated TTR fusions to two known SARS-CoV-2 CD4 EPs, S62-76 (VTWFHAIHVSGTNGT) and Orf3a 266-280 (EPIYDEPTTTTSVPL) (*30*). Two doses of TTR fusion immunizations elicited S62-specific cytokine responses of up to 2%, primarily consisting of IFN-γ^+^TNFα^+^ cells (0.85%) (fig. S3A). The response to Orf3a 266-280 encompassed a mix of IFN-γ, TNFα, and interleukin-2 (IL-2)–secreting CD4 T cells which totaled 7% of all blood CD4^+^ T cells (fig. S3A). To further characterize the CD4 T cell phenotype induced by TTR + c-di-GMP, we analyzed antigen-induced secretion of additional cytokines [IL-4, IL-10, IL-17, and transforming growth factor–β (TGFβ)] and transcription factor expression in antigen-specific CD4 T cells. We predominantly observed T-bet^+^ T helper 1 (T_H_1) CD4 T cells with minimal GATA-3^+^ T_H_2, RORγt^+^ T_H_17, and Foxp3^+^ regulatory T cells (T_reg_ cells) being induced (fig. S3, C and F). Similarly, we observed predominantly IFN-γ (T_H_1) secretion with antigen restimulation, with minimal IL-4 (T_H_2), IL-17 (T_H_17), IL-10, and TGFβ (T_reg_) detected (fig. S3, D and G). Thus, vaccinations with TTR-EP fusions together with c-di-GMP adjuvant primarily induced T_H_1 CD4 T cell responses.

To test whether combining CD8 and CD4 EPs affected the T cell response to each individual epitope, we immunized mice with TTR-S536-543, TTR-S259-267, and TTR-S62-76 TTR-peptide fusions individually or in combinations, where CD8 combo contains physical mixtures of TTR-S536 and TTR-S259 and CD4 + CD8 combo contains TTR-S536, TTR-S259, and TTR-S62 at amounts of 3 nmol each TTR fusion protein. The amount of each individual TTR-EP component was kept at 3 nmol per dose. Reassuringly, we detected T cell responses to all immunized EPs in the combinations (Fig. 2, H and I, and fig. S3B). As expected, immunization with CD8 combo TTR fusion vaccines elicited negligible spike-specific immunoglobulin G (IgG; Fig. 2J). When S536 and S259 were combined (CD8 combo), S536 response remained the same as S536 alone (Fig. 2H). However, the S259-specific response decreased by ~50% compared to S259 alone (8%) (Fig. 2I). When the CD4 epitope S62 was added to the combination (CD4 + CD8 combo), the CD8 T cell responses to either S536 or S259 were unaffected (Fig. 2, H and I). Similarly, the S62 response was not affected in the CD4 + CD8 combo compared to S62 alone (fig. S3B). Therefore, combining CD8 EPs led to a biased response toward the immunodominant epitopes, but CD4 responses did not affect and was not affected by the immunodominance of CD8 epitopes. Nonetheless, CD8 combo and CD4 + CD8 combo immunizations elicited T cell responses to each individual epitope, allowing us to subsequently evaluate the protective efficacy of T cell vaccines containing either individual or combinations of SARS-CoV-2 epitopes.

### Subcutaneous immunizations with SARS-CoV-2 CD8 T cell epitopes lowered lung viral load but failed to protect against high-dose SARS-CoV-2 challenge

To investigate whether vaccination with T cell epitopes alone is sufficient to control viral infection, we immunized K18-hACE2 mice subcutaneously with T cell EPs individually or in combinations. K18-hACE2 mice carry the human ACE2 transgene driven by the human keratin 18 promoter which directs expression to airway epithelia, among other tissues (*34*). The hACE2 transgene renders K18-hACE2 mice susceptible to infection by SARS-CoV-1 and SARS-CoV-2 strains (*35*). We selected two CD8 epitopes (BA1.S536-543 and BA1.S259-267) and one CD4 epitope (S62-76) for initial testing based on the following criteria: (i) sequence conservation in the USA-WA1/2020 SARS-CoV-2 strain available for viral challenge experiments, (ii) performance in the MHC-I stabilization assay (fig. S1), and (iii) immunogenicity as TTR fusion. As a negative control, we included Ova257-264 (SIINFEKL), an irrelevant CD8 T cell epitope. We additionally tested CD8 combo (S536 + S259) and CD4 + CD8 combo (S536 + S259 + S62).

**Fig. 3.**
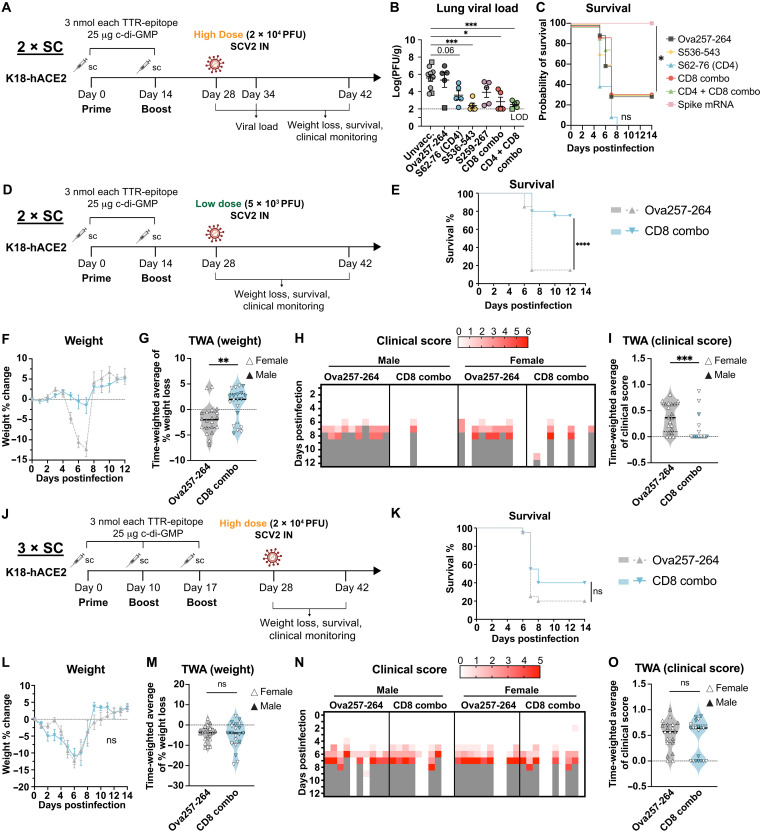
Subcutaneous immunization with SARS-CoV-2 CD8 T cell EPs conferred protection against low-dose but not high-dose SARS-CoV-2 challenge. (**A**) Female K18-hACE2 mice received immunizations and 2 × 10^4^ PFU of SARS-CoV-2 as indicated. CD8 combo, S536-543 and S259-267; CD4 + CD8 combo, S62-76, S536-543, S259-267. One microgram of intramuscular spike mRNA-LNP vaccine served as a positive control. (**B**) Lung viral load measured by log-transformed PFU per gram of lung tissue. Square points, mice found dead or euthanized before 6 dpi. Mean ± SEM is shown. One-way ANOVA and Tukey’s test. *N* = 10, unvaccinated; *N* = 5 for all other groups. (**C**) Survival. Log-rank Mantel-Cox test and Bonferroni correction. *N* = 10. (**D** to **I**) Low-dose SARS-CoV-2 challenge after 2 × SC immunizations. (D) K18-hACE2 mice received immunizations and 5 × 10^3^ PFU SARS-CoV-2 as indicated. *N* = 20 [10 males (M), 10 females (F)]. (E) Survival. Log-rank Mantel-Cox test. (F) Weight change. Mean ± SEM. (G) Violin plot shows time-weighted average (TWA) of weight change 1 to 12 dpi with median and quartiles. Mann-Whitney test. (H) Clinical score. Gray, animal found dead or euthanized. (I) Violin plot shows TWA of clinical scores with median and quartiles. Mann-Whitney test. (**J** to **O**) High-dose SARS-CoV-2 challenge after 3 × SC immunizations. (J) K18-hACE2 mice received immunizations and 2 × 10^4^ PFU SARS-CoV-2 as indicated. *N* = 20 (10 M, 10 F). (K) Survival. Log-rank Mantel-Cox test. (L) Weight change (mean ± SEM). (M) Violin plot shows TWA of weight change 1 to 12 dpi with median and quartiles. Mann-Whitney test. (N) Clinical score. (O) Violin plot shows TWA of clinical scores with median and quartiles. Mann-Whitney test. LOD, limit of detection.

Two weeks after the last immunization with single or combination of CD8 and CD4 EPs, mice were intranasally challenged with 2 × 10^4^ plaque-forming units (PFU) USA-WA1/2020 strain of SARS-CoV-2. Lung viral loads were quantified by plaque assay 6 days postinfection (dpi) (Fig. 3A). Compared to unvaccinated controls, S536-only immunization resulted in a ~1000-fold reduction in lung viral titers (Fig. 3B). S62-only immunization resulted in ~100-fold reduction in viral load (adjusted *P* = 0.06). While S259 showed a trend toward reduction in viral load, the results were not statistically significant (adjusted *P* = 0.22). As expected, immunization with Ova257-264, a non–SARS-CoV-2 epitope, failed to reduce viral load, indicating that SARS-CoV-2–specific T cells were required for protection. CD8 combo and CD4 + CD8 combo reduced lung viral titers to a similar degree as S536 alone, indicating a major contribution of S536-543 to viral clearance. Similar trends were observed when mice were challenged 6 months after the initial immunization (fig. S4A). At this later time point, S62, S536, and the CD8 combo groups continued to show reduced lung viral loads, while S259 immunization remained ineffective (fig. S4B). Notably, the CD4 + CD8 combo provided the most consistent long-term protection, achieving a nominal adjusted *P* value of 0.05. Together, these findings indicate that subcutaneous vaccination with individual SARS-CoV-2 T cell epitopes can significantly reduce lung viral burden and that such protection may persist for at least 6 months postimmunization.

To further gauge the impact of T cell EP vaccination on clinical outcomes and survival after infection, mice were challenged with 2 × 10^4^ PFU WA1/2020 and clinically monitored for 14 days. We focused on S536, S62, CD8 combo, and CD4 + CD8 combo based on their ability to lower lung viral load and included IM spike mRNA-LNP vaccination as a benchmark. While spike-mRNA conferred complete protection from mortality, S536, CD8 combo, nor CD4 + CD8 combo vaccination improved survival beyond Ova257-264 controls (30% survival) (Fig. 3C). Unexpectedly, all S62-immunized animals succumbed to disease, suggesting a potentially deleterious effect of this CD4 epitope alone (Fig. 3C). Likewise, none of the T cell EP vaccines (either alone or in combination) prevented weight loss or improved clinical scores, whereas spike mRNA vaccination completely protected against clinical symptoms (fig. S4, C and D). These results indicate that despite markedly reducing lung viral loads, two subcutaneous immunizations with T cell EPs are insufficient to confer clinical protection against SARS-CoV-2.

### Subcutaneous immunizations with SARS-CoV-2 CD8 T cell EPs were protective against low-dose SARS-CoV-2 challenge

Previous reports found that uncontrolled infection in the brain is the main cause of death in SARS-CoV-2 infections in K18-hACE2 mice (*36*, *37*). We reasoned that during high-dose infections, T cell response alone (without neutralizing antibodies) is insufficient for protection due to rapid viral spread into the brain where T cell surveillance is often impaired (*38*). However, at low dose, early control of viral infection in the respiratory tract by preexisting T cell response might possibly prevent brain dissemination and therefore lead to better disease outcomes. To test this hypothesis, we carried out the viral challenge with a lower viral dose (Fig. 3D) and focused on the CD8 combo vaccine which showed the greatest promise in viral load reduction. When the infectious dose was lowered to 5 × 10^3^ PFU, the CD8 combo vaccination led to significantly increased overall survival (75% versus 15%; Fig. 3E), reduction in weight loss (Fig. 3, F and G), and other clinical symptoms (Fig. 3, H and I). The protection was more pronounced in male mice compared to female mice (fig. S5). Thus, two subcutaneous immunizations with CD8 T cell EPs effectively controlled low-dose SARS-CoV-2 infection, and the protection was more pronounced in male mice compared to female mice.

We explored the possibility that a third vaccination of T cell EPs could protect against high-dose SARS-CoV-2 challenge (Fig. 3J). Mice were primed on day 0 and boosted on days 10 and 17 subcutaneously with TTR-EP fusions with c-di-GMP as adjuvant and challenged on day 28 with high-dose SARS-CoV-2 (2 × 10^4^ PFU). CD8 combo vaccination resulted in slightly increased survival compared to controls (40% versus 20%), but the difference was not statistically significant (Fig. 3M). Likewise, no reduction in weight loss (Fig. 3, L and M) or clinical scores (Fig. 3, N and O) was observed. Thus, a third subcutaneous immunization of CD8 T cell EPs was still insufficient for protection against high-dose SARS-CoV-2 challenge.

### IN boost strengthens mucosal T cell immunity and provides protection against high-dose SARS-CoV-2 challenge

**Fig. 4.**
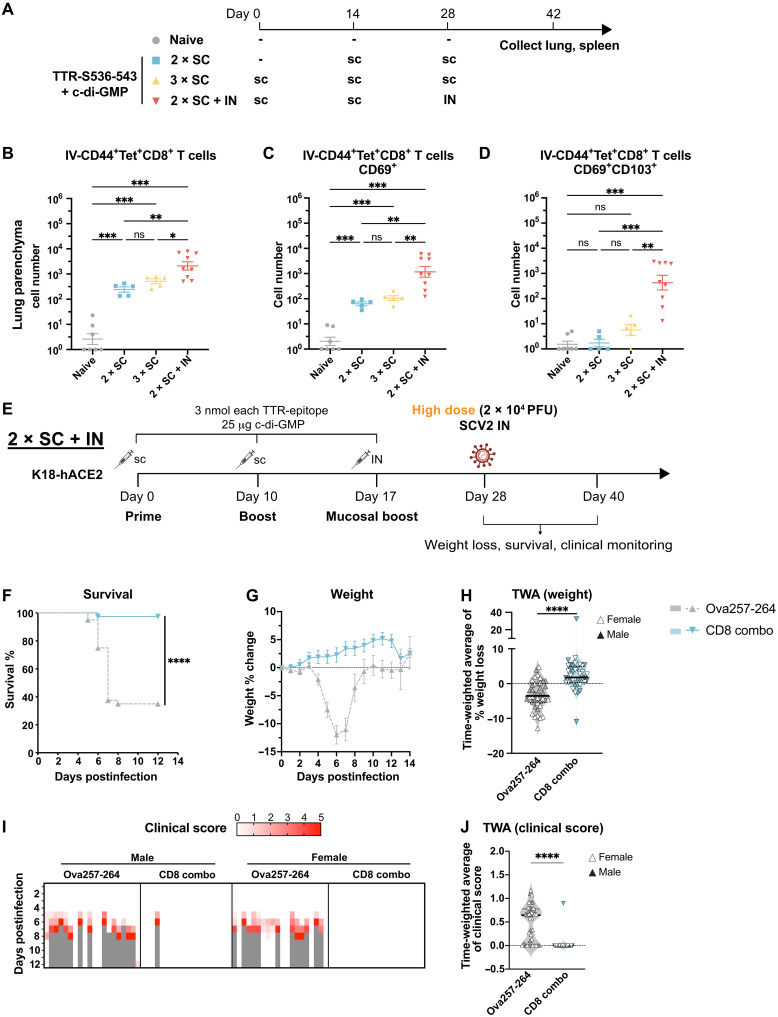
Adjuvanted intranasal boost strengthens mucosal T cell immunity and provides protection against high-dose SARS-CoV-2 challenge. (**A**) Female C57BL/6 mice received indicated immunizations. *N* = 7 for naïve, *n* = 5 for 2 × SC and 3 × SC and *n* = 9 for 2 × SC + IN. (**B** to **D**) S536-specific T cell response in the lung parenchyma on day 42 quantified as (B) CD45IV^−^ CD44^+^ S536-tetramer^+^, (C) CD69^+^ CD45IV^−^ CD44^+^ S536-tetramer^+^, and (D) CD69^+^ CD103^+^ CD45IV^−^ CD44^+^ S536-tetramer^+^ CD8^+^ T cells. Cell numbers per 10^5^ total lung cells were shown. (**E**) K18-hACE2 received indicated immunizations and 2 × 10^4^ PFU SARS-CoV-2 and monitored for 12 days. CD8 combo, S536-543 and S259-267. *N* = 39 for CD8 combo (19 M, 20 F). *N* = 40 for Ova257-264 (20 M, 20 F). Data were pooled from two independent experiments with similar results. (**F**) Survival. Log-rank Mantel-Cox test was performed. (**G** and **H**) Weight change. (G) Weight change. Mean ± SEM is shown. (H) Violin plot shows TWA of weight change calculated between 1 and 12 dpi. Filled and open symbols represent males and females, respectively. Median and quartiles are shown. Mann-Whitney test was performed. (**I**) Clinical scores. Gray box indicates that the animal was found dead or euthanized because of humanitarian endpoints. (**J**) Violin plot shows TWA of clinical scores. Filled and open symbols represent males and females, respectively. Median and quartiles are shown. Mann-Whitney test was performed.

Given the site of viral inoculation, we wondered whether IN boosting could have higher efficacy by enhancing mucosal T cell immunity in the respiratory tract. We first compared the systemic and lung T cell immune response after two subcutaneous immunizations (2 × SC), three subcutaneous immunizations (3 × SC), or two subcutaneous immunizations followed by an IN immunization (2 × SC + IN) of S536-543 (Fig. 4A). c-di-GMP was used as adjuvant for all immunizations. To distinguish cells in the lung parenchyma and cells in vasculature, we performed intravascular labeling with a CD45 antibody before lung tissue collection and analysis (Materials and Methods). Both 2 × SC and 3 × SC resulted in a detectable induction of CD44^+^ S536-tetramer^+^ CD8^+^ T cells in the lung parenchyma compared to naïve mice (Fig. 4, B to D). The 2 × SC + IN resulted in significantly more S536-tetramer^+^ CD8^+^ T cells in the lung compared to 2 × SC or 3 × SC (Fig. 4B). These cells also expressed higher percentages of traditional resident memory T cells (T_RM_) markers, CD69 and CD103 (Fig. 4D). The systemic T cell response, as measured by CD44^+^ S536-tetramer^+^% of CD8 T cells in the spleen, was similar among 2 × SC, 3 × SC, and 2 × SC + IN (fig. S7A). Thus, a third IN immunization with adjuvant significantly boosts mucosal T cell immunity evidenced by high numbers of antigen-specific T_RM_ cells in the lung.

To determine whether the strong mucosal T cell response induced by IN boost translates into better clinical protection, mice were immunized with CD8 combo (S536 + S259) on days 0 and 10 subcutaneously, boosted on day 17 intranasally, and challenged with high-dose (2 × 10^4^ PFU) SARS-CoV-2 (Fig. 4E). The CD8 combo vaccination significantly improved survival compared to Ova257-264 control (97.4% versus 35%) (Fig. 4F). Virtually no weight loss was observed in CD8 combo vaccinated mice (Fig. 4, G and H). Similarly, virtually no clinical symptom developed in CD8 combo vaccinated mice, except 1 of 19 male mice (Fig. 4, I and J). Notably, this level of protection from clinical symptoms is comparable to spike mRNA vaccines which elicit both antibody and T cell–mediated protection (Fig. 3C and fig. S4). No difference in protection was observed between male and female mice (fig. S6). Thus, a third IN boost with CD8 EPs provides potent protection against high-dose SARS-CoV-2 challenge at a comparable level to spike mRNA vaccines.

### AF IN boost elicits equally potent mucosal T cell immunity and provides protection against high-dose SARS-CoV-2 challenge

IN delivery of vaccines offers a promising approach for preventing respiratory infections. However, clinical applications have encountered challenges due to adjuvant-induced inflammation, leading to adverse reactions (*21*, *22*, *26*). To address these limitations, we investigated whether IN administration of the TTR-EP alone (AF) could elicit mucosal T cell immunity and protect against high-dose SARS-CoV-2 infection.

**Fig. 5.**
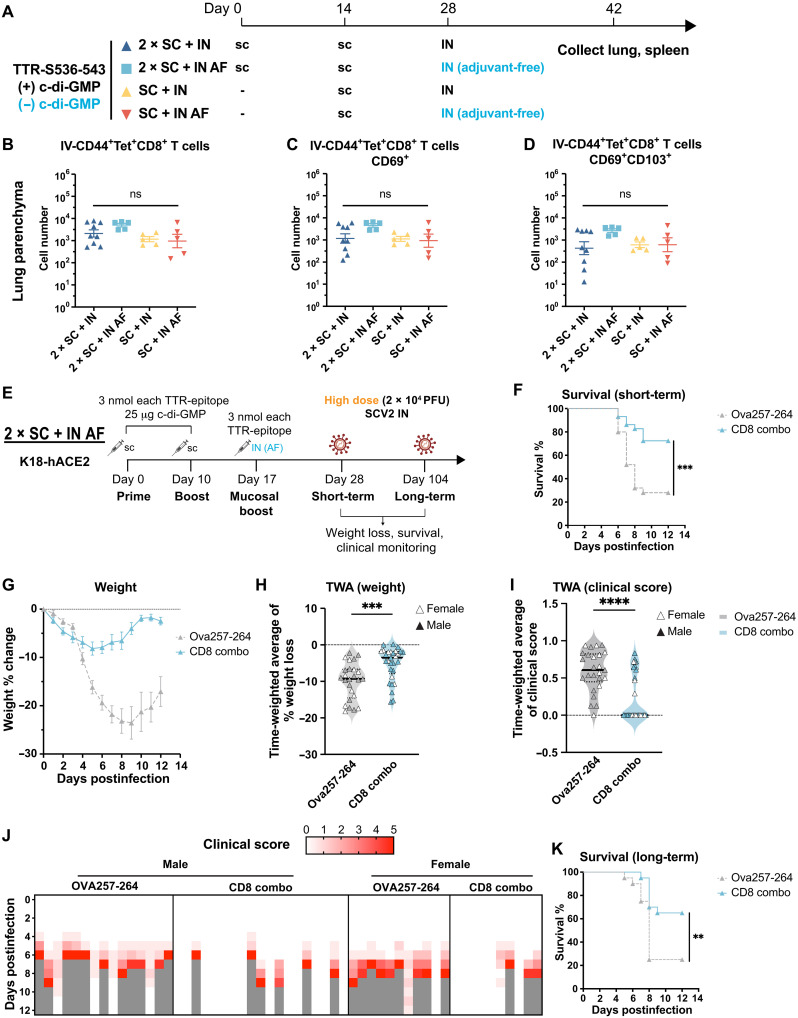
AF intranasal boost elicits equally potent mucosal T cell immunity and provides protection against high-dose SARS-CoV-2 challenge. (**A**) Female C57BL/6 mice received indicated immunizations. *N* = 9 for 2 × SC + IN, *n* = 5 for other groups. (**B** to **D**) S536-specific T cell response in the lung parenchyma on day 42 quantified as (B) CD45IV^−^ CD44^+^ S536-tetramer^+^, (C) CD69^+^ CD45IV^−^ CD44^+^ S536-tetramer^+^, and (D) CD69^+^ CD103^+^ CD45IV^−^ CD44^+^ S536-tetramer^+^ CD8^+^ T cells. Cell numbers per 10^5^ total lung cells were shown. (**E**) K18-hACE2 received indicated immunizations and 2 × 10^4^ PFU SARS-CoV-2 on day 28 (short term) or day 104 (long term) and monitored for 12 days. For short-term study, *N* = 25 for Ova257-264 (15 M, 10 F). *N* = 29 for CD8 combo (19 M, 10 F). Data were pooled from two independent experiments with similar results. For long-term study, *N* = 20 (10 M, 10 F) for both Ova257-264 and CD8 combo groups. (**F** to **J**) Data from short-term study (day 28). (F) Weight change shown as mean ± SEM. (G) Violin plot shows TWA of weight change calculated between 1 and 12 dpi. Filled and open symbols represent males and females, respectively. Median and quartiles are shown. Mann-Whitney test was performed. (H) Survival. Log-rank Mantel-Cox test. (I) Clinical scores. Gray box indicates that the animal was found dead or euthanized because of humanitarian endpoints. (J) Violin plot shows TWA of clinical scores. Filled and open symbols represent males and females, respectively. Median and quartiles are shown. Mann-Whitney test was performed. (**K**) Survival data from long-term study (day 104). Log-rank Mantel-Cox test.

To assess the ability to induce mucosal T cell response, we performed IN boosts with or without adjuvant after either one or two doses of subcutaneous immunizations with adjuvant using the TTR S536-543 antigen (Fig. 5A). We found comparable numbers of CD44^+^ S536-tetramer^+^ CD8^+^ T cells in the lung parenchyma regardless of adjuvant inclusion and number of subcutaneous doses (Fig. 5, B to D). CD69 and CD103 expression is similar between AF IN and adjuvanted IN, suggesting intact T_RM_ formation even without IN adjuvant. Notably, the lung T cell response was not significantly influenced by one or two subcutaneous immunizations before the IN boost. In contrast, the systemic T cell response (assessed in the spleen) was markedly higher in groups receiving two versus one subcutaneous immunizations (fig. S7B). The presence of IN adjuvant did not affect spleen T cell responses regardless of one or two subcutaneous doses (fig. S7B). Thus, AF IN boost with TTR-EP fusions can induce equally robust lung-resident and systemic T cell immunity as adjuvanted IN boost.

We predicted that an AF IN booster could provide similar protection against high-dose SARS-CoV-2 given similar mucosal T cell responses with or without IN adjuvant (Fig. 5E). Eliminating the use of IN adjuvant is desirable for clinical translation as it can avoid adjuvant-induced toxicity and safety concerns from excessive inflammation in the airways (*20*, *21*). In a short-term viral challenge (Fig. 5E), 2 × SC + IN AF immunizations significantly protected mice from mortality compared to Ova257-264 controls during high-dose SARS-CoV-2 challenge (72% versus 28%; Fig. 5F). Slightly higher survival rates were observed in female mice (89% versus 65%; fig. S8C). Similarly, CD8 combo led to reduced weight loss (Fig. 5, G and H) and clinical score compared to controls (Fig. 5, I and J). Weight and clinical scores were similar between male and female mice (fig. S8). Notably, a small degree of weight loss was observed before 6 dpi followed by gradual rebound (Fig. 5G). In contrast, adjuvanted IN boost resulted in a slow and gradual body weight increase during SARS-CoV-2 challenge (Fig. 4F), suggesting that the inclusion of adjuvant during IN immunization provided more protection than AF immunization. Nonetheless, AF IN boosting with TTR-EP fusions is a promising strategy for inducing mucosal T cell immunity and provides potent protection against high-dose SARS-CoV-2 infection.

### IN T cell vaccine elicits durable protection against SARS-CoV-2

Last, we assessed whether 2 × SC + IN AF vaccination with the CD8 combo conferred long-term protection by conducting a high-dose SARS-CoV-2 challenge 104 days after the initial immunization (Fig. 5E). We first measured the frequency of antigen-specific T_RM_ cells still present in the lung parenchyma at this time point (fig. S10A). Compared to 2 × SC and 3 × SC, 2 × SC + IN AF vaccinations led to higher numbers of S536-specific CD69^+^ or CD69^+^CD103^+^ lung T_RM_ cells, similar to 2 × SC + IN with adjuvant (fig. S10, B to D). However, the number of CD69^+^ and CD69^+^CD103^+^ cells decreased about 10-fold compared to the earlier time point (Fig. 5, B to D). During high-dose viral challenge, CD8 combo–vaccinated mice (2 × SC + IN AF) exhibited improved survival even at this extended time point (65% versus 25% in controls; Fig. 5K). Clinical symptoms were also significantly attenuated in CD8 combo–vaccinated mice (fig. S11, A, D, and E), although no significant protection against weight loss was observed (fig. S11, B and C). These results suggest that while the number of lung T_RM_ cells and protection against virus-induced weight loss wane over time, substantial protection against mortality and clinical disease is largely maintained with 2 × SC + IN AF vaccinations of CD8 EPs.

## DISCUSSION

The emergence of SARS-CoV-2 and its subsequent variants necessitates development of vaccines that provide effective and durable protection for respiratory viral infections. While spike protein–based mRNA vaccines have been highly efficacious in preventing and reducing COVID-19 disease, both waning neutralizing antibody titers and loss of neutralizing activity toward antigenically divergent variants since the Omicron lineage contributed to reduced efficacy (*39*–*43*). Hence, repeated boosters that match circulating variants are required for continued immune protection. Increasing evidence suggests an essential role of cellular immunity in providing protection against COVID-19 (*6*–*8*, *44*–*46*). T cell immunity can be more durable and resistant to evasion by emerging variants (*9*, *12*, *47*–*49*). Thus, targeting conserved T cell epitopes is a promising strategy to achieve broad and durable protection against SARS-CoV-2 variants and potentially related coronaviruses (*14*, *16*, *50*). In this study, we adopted an epitope peptide–based T cell vaccine design to determine the protective effects of selected CD8 and CD4 T cell epitopes and combinations. This strategy allows the selective inclusion of conserved, protective T cell epitopes in vaccines and reduces the risk of the overall response being dominated by nonprotective epitopes which can be introduced in vaccines that contain long segments or full-length antigens.

We first mapped the T cell epitope landscape in Omicron BA.1 spike protein in mice to investigate differences in the epitope landscape between Wuhan and BA.1. We observed that two of the most immunodominant epitopes, BA1.S536-543 and BA1.S259-267, are conserved with Wuhan (*30*), suggesting that the immunodominance hierarchy remained relatively unchanged in BA.1 with respect to these MHCs and that T cell immunity is potentially cross-protective toward Wuhan and BA.1. Notably, several BA.1 epitopes differed in sequence compared to Wuhan, such as BA1.S971-981 and BA1.S486-494, suggesting the presence of variant-specific T cell immunity. One avenue where these epitopes could prove useful is tracking BA.1-specific responses in different immunological settings or vaccination regimens (e.g., heterologous boosting). Such studies could help elucidate mechanisms of immunodominance and inspire strategies for broadening T cell responses to viral variants of interest.

Development of effective T cell–directed vaccines has been hindered by the poor immunogenicity of peptides. TTR is a pharmacokinetically appropriate carrier protein that improves immunogenicity through (i) increased lymph node retention and (ii) the increased rate of systemic clearance (*33*). Increased lymph node retention allows more efficient antigen capture and presentation by antigen-presenting cells in the draining lymph node, which are concurrently activated by coadministered adjuvants. We observed excellent immunogenicity for multiple SARS-CoV-2 CD8 and CD4 T cell EPs. CD8 T cell responses induced by TTR fusions together with c-di-GMP were polyfunctional, capable of secreting IFN-γ and TNFα in addition to degranulation. The CD4 responses induced this way were almost exclusively T_H_1 polarized, capable of secreting IFN-γ, TNFα, and IL-2.

The importance of strong mucosal humoral and cellular immunity in protecting against SARS-CoV-2 and other respiratory viruses is increasingly evident (*51*, *52*). However, the relative necessity of mucosal T cell or antibody immunity for enhanced protection remained unclear. Our findings offer several insights. First, IM spike mRNA vaccines, which primarily elicit systemic antibody and T cell responses, conferred complete protection against high-dose SARS-CoV-2 infection, consistent with previous reports (*15*, *53*). As CD8 depletion did not lead to loss of protection (*15*, *53*), the systemic antibody response is the major driver of protection by IM spike mRNA vaccine, and systemic antibody response alone is sufficient for protection. Second, we evaluated protection provided by CD8 T cell EP vaccination alone, without the generation of SARS-CoV-2–specific antibodies. While subcutaneous vaccinations with CD8 T cell EPs protected mice from low-dose (5 × 10^3^ PFU) SARS-CoV-2 infection, it failed to protect against high-dose (2 × 10^4^ PFU) infection despite substantially lowering lung viral load. Our findings are consistent with Pardieck *et al.* (*14*)’s report where subcutaneous vaccinations with an SLP containing the S536-543 epitope (Wuhan-1 S539-546) protected mice from 5 × 10^3^ PFU (low-dose) SARS-CoV-2 challenge. Unlike our results, their vaccinated mice experienced substantial weight loss, with most approaching the 20% humane euthanasia threshold. Protection also required three doses of the SLP vaccine, each containing 100 μg of peptide, while our TTR fusion vaccines not only significantly improved survival but also minimized weight loss following challenge with the same viral dose, indicating superior protection. Notably, each dose of the TTR fusion vaccine contained a 13-fold lower molar antigen amount than the SLP vaccine, further indicating enhanced immunogenicity. Notably, whether the SLP vaccine could protect against a higher viral dose was not examined. Our data indicate that only IN boosting ensured clinical protection against high-dose infection, whereas even three doses of the subcutaneous TTR fusion vaccine did not. Collectively, our results indicate that for low-dose infections, both systemic antibody and systemic T cell responses suffice for complete protection. However, for high-dose viral infections, systemic antibodies protect, but T cells require IN immunization for protection. This underscores the importance of inducing mucosal T cell immunity to augment T cell–mediated protection, particularly in scenarios of viral variant–driven antibody escape, and emphasizes the need for airway-targeted vaccines to achieve more robust and durable protection.

One attractive feature of mucosal immunity is the ability to decrease viral shedding and transmission (*54*, *55*). This was demonstrated in the hamster model by Mao *et al.* (*26*) using a “prime and pull” vaccination strategy which consisted of IM prime with spike mRNA and an unadjuvanted IN boost with recombinant spike protein. This induced strong mucosal antibody and T cell responses and led to decreased transmission in cohoused hamsters. However, the relative contribution of antibody and T cell responses in that scenario still requires further investigation. As SARS-CoV-2 variants such as Omicron develop heightened transmissibility, it would be of great interest to determine whether systemic or mucosal T cell responses to conserved epitopes is capable of reducing transmission of various variants.

While current IM administered COVID-19 vaccines demonstrated efficacy toward the early SARS-CoV-2 variants, their limited ability to induce mucosal immunity compared to natural infection (*17*) may hinder their potency against emerging variants. In this study, we showed that IN delivery of TTR-EP fusions induced strong lung-resident T cell responses in mice previously immunized with the same EP parenterally. Notably, the IN boost did not require adjuvant for lung T_RM_ formation, suggesting that simply the presence of antigen in the respiratory mucosa could convert parenterally primed memory T cell responses into lung T_RM_ cells. Our findings corroborate with the prime and pull vaccination strategy taken by Mao *et al.* (*26*), which showed robust lung T_RM_ formation after IM prime with spike mRNA-LNP followed by IN boost with unadjuvanted recombinant spike protein. This mucosal antigen recall response could arise from the local expansion of preexisting T_RM_ cells (*56*) or the recruitment of circulating T_EM_ cells. Given safety concerns associated with the use of mucosal adjuvants (*21*, *57*, *58*) and that a substantial portion of the global population has been primed to SARS-CoV-2 through IM vaccinations, AF IN antigen boosting presents an appealing avenue for enhancing T cell–mediated mucosal immunity for SARS-CoV-2 and potentially other respiratory viruses.

One limitation of the current study is that the K18-hACE2 mouse model might be an imperfect model for human SARS-CoV-2 infection. It was suggested that neuroinvasion of SARS-CoV-2, rather than lung viremia, is the major cause of mortality in the K18-hACE2 model (*36*, *37*). In human, COVID-19 is primarily a respiratory disease, and direct infection of the central nervous system is not considered a major cause of death. However, some reports have described COVID-19–associated neurological symptoms (*59*), and SARS-CoV-2 has been detected in human brain cortical neurons postmortem (*60*), suggesting a role of neuroinvasion in some COVID-19 pathologies. In this respect, our findings in the K18-hACE2 model suggest that mucosal T cell immunity might provide protection against respiratory and neural pathologies through early clearance of respiratory tract infection. Future studies should evaluate T cell vaccines in hamster and macaque models which more closely reflect human COVID-19 infections.

In summary, we developed highly immunogenic carrier-protein fusion T cell vaccines and showed that as few as one or two CD8 T cell epitopes can provide potent and durable protection against SARS-CoV-2 which is further enhanced by IN boosting. Future research should focus on incorporating additional T cell epitopes from diverse viral proteins to improve efficacy and mitigate the risk of mutational immune escape. While the diversity of human MHC-I limits the feasibility of universal vaccine epitopes, it is possible to computationally design a collection of epitopes that achieves broad population coverage. Our results demonstrate the potential of conserved T cell epitope peptide–based vaccines combined with mucosal immunization to provide broad and long-lasting immunity against SARS-CoV-2 and related coronaviruses in the wake of ongoing viral evolution and other emerging viral threats.

## MATERIALS AND METHODS

### Experimental design

The objectives of the research were to identify conserved T cell epitopes, develop carrier protein-fusion antigens that elicit strong T cell responses exclusively, and determine whether T cell–inducing vaccines alone provide protection against SARS-CoV-2 challenge in mouse models. The first goal of the study is to determine the CD8 T cell epitope landscape of Omicron BA.1 variant in C57BL/6 mice. To ensure reproducibility and confidence, the epitope mapping experiment was replicated four times with sex-balanced groups of animals with an optimized the protocol that achieves high sensitivity and specificity. To avoid biased assessments of clinical symptoms, researchers and animal handlers were blinded during the SARS-CoV-2 challenge studies. Researchers were not blinded during quantitative assays. Animals were randomly assigned to experimental groups. Sample sizes were empirically chosen on the basis of preliminary experiments.

### Ethics statement

All animal procedures were in compliance with the Office of Laboratory Animal Welfare and the National Institutes of Health (NIH) Guide for the Care and Use of Laboratory Animals. All procedures were approved by Institutional Animal Care and Use Committees (IACUCs) and Institutional Review Boards (IRBs) in their respective institutions. Part of the study was performed at the Center for Comparative Medicine in Brigham and Women’s Hospital (protocol 2018N000188). Part of the study was performed at the University of Missouri’s Laboratory for Infectious Disease Research (LIDR) Biosafety Level 3 (BSL3) facility (protocol 43226 Amendment 4.2). Biohazardous procedures were approved by the University of Missouri Institutional Biosafety Committee, and all work involving SARS-CoV-2 was conducted at the LIDR under BSL3/Animal Biosafety Level 3 (ABSL3) conditions. Part of the study was performed in the biosafety level 4 laboratory of the National Emerging Infectious Diseases Laboratories (NEIDL) of Boston University (protocol 202000038). Both LIDR BSL3 and NEIDL BSL4 facilities are Association for the Assessment and Accreditation of Laboratory Animal Care accredited.

### Mice

C57BL/6J and B6.Cg-Tg(K18-ACE2)2Prlmn/J (K18-hACE2) mice (stock no. 034860) were purchased from the Jackson Laboratory (*34*, *35*). Upon receipt into the LIDR from Brigham and Women’s Hospital, animals were inspected, and no health issues were noted. Animals were allowed to acclimate before any immunizations or viral challenge. Mice aged 6 to 14 weeks were used at the beginning of immunizations.

### Cell lines

Expi293F cells (Thermo Fisher Scientific, catalog no. A14527) were cultured and transfected according to the manufacturers’ instructions, using the Expi293 Expression System Kit (Thermo Fisher Scientific, catalog no. A14635). Briefly, Expi293F cells were maintained in a 37°C incubator with ≥80% relative humidity and 8% CO_2_ on an orbital shaker platform at a shake speed of 125 rpm. Cells were subcultured when densities reached 3 to 4 × 10^−6^/ml. Human embryonic kidney (HEK) 293T cells and HEK293T-derived EpiScan cells were maintained in Dulbecco’s modified Eagle’s medium (DMEM) supplemented with 10% fetal bovine serum (FBS) and 1× penicillin-streptomycin. *Cercopithecus aethiops* kidney cells [Vero E6; American Type Culture Collection (ATCC), catalog no. CRL-1586] were maintained in high-glucose DMEM (catalog no. 11965092, Gibco) supplemented with 10% Serum Plus II FBS substitute (Millipore Sigma, catalog no. 14009C) and GlutaMAX (Thermo Fisher Scientific, catalog no. 35050061) at 37°C with 5% CO_2_.

### Peptides

Peptide libraries for BA.1 spike were synthesized as crude peptides by GenScript. Peptides were dissolved in dimethyl sulfoxide (DMSO) to a stock concentration of 10 mg/ml and further diluted to a working concentration of 100 μg/ml with Dulbecco’s phosphate-buffered saline (DPBS). Other peptides were synthesized with >80% purity by GenScript and dissolved with appropriate solvents and concentrations, typically DMSO at 10 mg/ml.

### Lentiviral production

HEK293T or HEK293FT cells were transfected with lentiviral transfer plasmid and packaging plasmids pMD2.G (Addgene, catalog no. 12259) and psPAX2 (Addgene, catalog no. 12260) using PolyJet In Vitro DNA Transfection Reagent (SignaGen, catalog no. SL100688) according to the manufacturer’s protocol. Media was replaced 16 hours after transfection. Viral supernatants were collected 48 and 72 hours after transfection, combined, passed through a 0.45-μm filter, and concentrated using a Lenti-X concentrator (Takara, catalog no. 631232).

### EpiScan cells and cell-based pMHC binding assay

MHC-I–, TAP1/TAP2-, and ERAP1/ERAP2-null EpiScan cells were generated in (*31*). Mouse H-2K^b^ and H-2D^b^ alleles were synthesized as gBlocks by Integrated DNA Technologies (IDT) and cloned into pHAGE-EF1a-DEST-PGK-Bsd lentiviral vectors. H-2K^b^ + or H-2D^b^ + EpiScan cells were generated with lentiviral transduction followed by selection in blasticidin media (10 μg/ml). To measure peptide-MHC (pMHC) binding, 100,000 H-2K^b^ + or H-2D^b^ + EpiScan cells were plated in 96-well flat-bottom plates with serum-free DMEM. Plates were precoated with cDMEM for 10 min, and the media was discarded before cell plating. Peptides were added at 0.1, 1, 10, and 100 μM final concentrations. DMSO only and irrelevant peptides were used as negative controls. After overnight incubation in the 37°C incubator, cells were collected by pipetting and transferred to 96-well U-bottom plates for flow cytometry staining. The cells were washed two times with DPBS, collected by 500*g* 5-min centrifugation, then stained with anti-human β2m antibody (BioLegend, catalog no. 154506) and Zombie Aqua Fxiable Viability Dye (BioLegend, catalog no. 423101) for 20 min at room temperature, washed two times with DPBS, and resuspended in phosphate-buffered saline (PBS) for acquisition. Mean fluorescent intensities of β2m staining were reported. Median effective concentration values were calculated by fitting data to three parameter dose-response curve in GraphPad Prism 10.

### Immunizations

#### Epitope mapping with Omicron BA.1 spike protein

Omicron BA.1 spike protein was produced as described in (*32*). Eight to 10 (half male, half female) C57BL/6J mice were used for each replicate of epitope mapping experiments. For prime immunizations, mice were anesthetized with isoflurane and injected subcutaneously with 100 μg of Omicron BA.1 spike protein and 25 μg of c-di-GMP (Invitrogen, catalog no. tlrl-nacdg) at both sides of the tail base. Fourteen days later, mice were boosted with the same amount of antigen and adjuvant. Half the volume was injected at back of the neck, and the other half was injected at both sides of the tail base. On day 21, mice were euthanized. Spleens and lymph nodes were pooled and processed into single-cell suspensions and subsequently used for CD8 T cell isolation.

#### TTR-EP fusion immunizations

Subcutaneous injections were performed at both sides of the tail base. For IN injections, indicated amounts of TTR protein and adjuvant were diluted in 30 μl of DPBS and installed equally into each nostril of the mouse with a P20 pipette under isoflurane anesthesia. Unless otherwise stated, 3 nmol of TTR fusion protein and 25 μg of c-di-GMP were dosed per injection, similar to Mehta *et al.* (*33*). A total of 3 nmol of the TTR-EP fusion is equivalent to about 3 μg of the free peptide and is in range with other peptide vaccine studies (*61*–*63*). In cases where combinations were used for immunizations, the combination consisted of physical mixtures of individual TTR-EP fusion proteins, where each component was dosed at the indicated amount (e.g., 3 nmol of CD8 combo vaccine consisted of 3 nmol of TTR-S536 and 3 nmol of TTR-S259).

### ELISPOT

CD8^+^ T cells were isolated through negative selection from the spleen and lymph nodes using the Miltenyi CD8a^+^ T cell Isolation Kit, mouse (catalog no. 130-104-075) according to the manufacturer’s instructions. IFN-γ ELISPOT was performed using the BD ELISPOT Mouse IFN-γ ELISPOT Pair (BD, catalog no. 551881) and MultiScreen HTS Filter Plates (Milipore, catalog no. MSIPS4W10). Plates were activated with 50 μl of 70% ethanol for <1 min, washed three times with DPBS, and coated with 100 μl of capture antibody (2 μg/ml) in DPBS overnight at 4°C. The plate was washed with DPBS and blocked with 200 μl of complete Click’s medium [Eagle Hanks’ Amino Acid(EHAA), Irvine Scientific, catalog no. 9195-500ML] for at least 30 min. A total of 250,000 isolated CD8 T cells were cocultured with 250,000 irradiated (25 Gy) splenocytes isolated from naïve mice (to serve as antigen-presenting cells) in the presence of peptide (10 μg/ml) in complete Eagle Hanks’ Amino Acid medium (cEHAA). The plate was placed in a 37°C incubator overnight (16 hours) undisturbed.

The next day, the cells were discarded, and the plate was washed two times with water and three times with 1× Phosphate-buffered saline with 0.05% Tween-20 (PBST). One hundred microliters of detection antibody diluted 1:100 in dilution buffer (DPBS + 10% FBS) was added to each well and incubated for 2 hours at room temperature. After three washes with 1× PBST, 100 μl of streptavidin–horseradish peroxidase in dilution buffer was added to each well and incubated for 1 hour at room temperature. The plates were washed four times with 1× PBST and two times with PBS. One hundred microliters of 3-amino-9-ethylcarbazole (AEC) substrate solution (BD, catalog no. 551951) was added. The reaction was stopped after 5 to 10 min (or until spots were visible) by washing wells with water three times. The plate was air-dried for 1 to 2 days, and spots were enumerated on a Mabtech IRIS 2 instrument.

### Enzyme-linked immunosorbent assay

Maxisorp plates (Thermo Fisher Scientific, 439454) were coated with SARS-CoV-2 Spike protein (GenScript, Z03481) at 0.5 μg/ml in PBS at 4°C overnight. After discarding the antigen solution, plates were blocked with 3% bovine serum albumin (BSA) PBS for 1 hour at room temperature. Plates were washed with 1× PBST (0.05% Tween 20). Sera were diluted at 1:50, 1:250, 1:1250, 1:6250, and 1:31250 with 1% BSA in 1× PBST (0.05% Tween 20) and added to plates. Plates were incubated at 4°C overnight. Plates were washed three times with 1× PBST (0.05% Tween 20). Alkaline phosphatase (AP)-conjugated goat anti-mouse IgG secondary antibody (Southern Biotech, 1030-04) diluted 1:1000 with 1% BSA in PBST (0.05% Tween 20) was added for 1.5 hours at room temperature. Plates were washed three times with 1× PBST (0.05% Tween 20). Two tablets of phosphatase substrate (Sigma-Aldrich, S0942) were dissolved in AP substrate buffer [0.1 M glycine (pH 10.4), with 1 mM MgCl_2_ and 1 mM ZnCl_2_], and 100 μl of substrate solution was added to each well. Plates were allowed to develop for 1 to 2 hours at room temperature followed by absorbance reading at 405 nm.

### TTR fusion protein expression and purification

His-tagged, codon-optimized mouse TTR-EP fusion proteins were cloned into pcDNA3.4 expression vectors using Golden Gate Assembly and transformed into DH5α-competent cells. Plasmids were prepared with low-endotoxin miniprep or midiprep kits (Zymo, catalog nos. D4200 and D4210). Maintenance of and protein expression in Expi293F cells were performed according to the manufacturer’s instructions. For transfection, 25 μg of DNA was used to transfect 75 × 10^6^ Expi293F cells at a density of 3 × 10^6^/ml using the ExpiFectamine 293 Transfection Kit (Thermo Fisher Scientific, A14524). The culture supernatant was collected 5 to 7 days posttransfection by centrifugation at 4000g for 30 min at 4°C.

Purification of His-tagged protein was done using TALON metal affinity resin (Takara, catalog no. 635504). The resin was washed twice with TALON equilibration buffer, combined with the clarified culture supernatant and incubated at 4°C for 2 to 3 hours with agitation. The resin was collected by centrifugation at 700*g* for 5 min, transferred to a gravity column, and washed three times with 5 bed volumes of equilibration buffer. The protein was eluted with 5 bed volumes of elution buffer (equilibration buffer with 150 mM imidazole) collected in one fraction. Buffer exchange was done using Amicon ultracentrifugation filters (30 kDa; Milipore Sigma, catalog no. UFC8030) with a total of four exchanges into DPBS. The final protein sample was sterilized by passing through 0.22-μm membrane filters (Costar Spin-X, catalog no. 8160). Protein concentrations were calculated from absorbance at 280 nm (A280) measurements using NanoDrop. Protein purity was assessed by denaturing SDS–polyacrylamide gel electrophoresis. Proteins were aliquoted and flash-frozen in liquid nitrogen and stored at −80°C until use. Thawed proteins were kept at 4°C and used within 1 month. TTR fusion antigen sequences are listed in table S2.

### Flow cytometry

All flow cytometry reagents are listed in table S3.

#### Preparation of single-cell suspensions

*Blood*One hundred to 200 μl of blood was lysed in 1.5 ml of Ammonium–chloride–potassium (ACK) lysis buffer for 5 min at room temperature. If necessary, a second lysis was done to further lyse residual red blood cells (RBCs). The remaining cells were pelleted at 300*g* for 5 min and resuspended in fluorescence-activated cell sorting (FACS) buffer (DPBS with 2% FBS) for antibody staining or cRPMI medium for stimulation.

*Spleen*The spleen was crushed using the back of the syringe against a 70-μm cell strainer. After RBC lysis, splenocytes were washed and resuspended with FACS buffer.

*Lung*To label intravascular cells (to distinguish from parenchymal cells), 1 μg of CD45 antibody (BV785) was injected retro-orbitally 3 to 5 min before mice were euthanized by CO_2_. Excised lungs were dissociated using the Lung Dissociation Kit, mouse (Miltenyi, catalog no. 130-095-927) and a gentleMACS dissociator. The cells were passed through a 70-μm strainer and subjected to RBC lysis by ACK lysing buffer (Gibco). To remove debris, cells were resuspended in 30% isotonic Percoll and centrifuged at 1500*g* for 30 min at room temperature. The debris and buffer were aspirated, and the pellet was resuspended with FACS buffer for flow cytometry staining.

#### Tetramer staining

The S536-543 (H-2K^b^) tetramer was obtained from the NIH Tetramer Core Facility. Single-cell suspensions were treated with FcR block for at least 5 min at room temperature in 96-well U-bottom plates. Tetramers were added at 1:50 dilution in the presence of 50 nM dasatinib (Axon Medchem, catalog no. 1392) for 60 min at 4°C protected from light. The remaining surface staining antibody cocktail (including the Fixable Viability Dye) was added for another 30 min at 4°C. If there were more than one Brilliant Violet (BV) fluorophores in the panel, then 5 μl of Brilliant Stain Buffer (BD, catalog no. 566385) was added into the antibody cocktail before mixing BV dyes. The cells were washed two times with additions of 200 μl of FACS buffer and 5-min 300*g* centrifugation steps in between washes. The cells were resuspended in FACS buffer for acquisition on the flow cytometer or in intracellular (IC) Fixation buffer containing 4% paraformaldehyde for acquisition at a later time or intracellular staining.

#### ICS and transcription factor staining

To stimulate lymphocytes with cognate peptide, RBC-lysed single cells derived from 100 to 200 μl of blood were resuspended in 100 μl of cRPMI media in 96-well U-bottom plates. Depending on the properties of the peptide, 1 to 10 μM peptide was added. Typically for a strong antigen such as Ova257-264, the maximal signal is achieved at 1 μM. For weaker antigens, 10 μM might be needed. In some cases, phorbol 12-myristate 13-acetate and ionomycin (eBioscience Cell Stimulation Cocktail, catalog no. 00-4970-93) was used for stimulation. After incubation at 37°C for 2 hours, brefeldin A (BioLegend, catalog no. 420601) was added at 1× concentration, and the cells were returned to the 37°C incubator for another 4 hours. At the end of stimulation, cells were washed one time with FACS buffer. FcR block was performed for at least 5 min at room temperature. Then, Fixable Viability Dye and the surface antibody cocktail were added for 30 min at 4°C protected from light. The cells were washed two times with additions of 200 μl of FACS buffer, with 5-min 300*g* centrifugation in between washes. Fixation and permeabilization were done according to the manufacturers’ instructions (eBioscience Intracellular Fixation and Permeabilization Buffer Set, Thermo Fisher Scientific, catalog no. 88-8824-00). Cells were resuspended in 100 μl of fixation buffer for 20 min at room temperature, followed by addition of 1× permeabilization buffer. After centrifugation at 600*g* for 5 min, cells were resuspended in 200 μl of 1× permeabilization buffer and centrifuged again. Intracellular antibodies to cytokines were diluted in 1× permeabilization buffer, and the cocktail was used to resuspend the cell pellet. The intracellular staining was carried out at room temperature for 30 min. The cells were washed two times with 1× permeabilization buffer, with centrifugation at 600*g* for 5 min. The washed cells were resuspended in FACS buffer for acquisition. Transcription factor staining was carried out using the eBioscience Foxp3/Transcription Factor Staining Buffer Set according to the manufacturer’s instructions. After viability and surface staining, cells were resuspended in 200 μl Fix/Perm buffer for 30 to 60 min at room temperature. Cells were pelleted at 600*g* for 5 min and resuspended in 200 μl of permeabilization buffer. Cells were washed again in permeabilization buffer and pelleted. Cells were then resuspended with 100 μl of permeabilization buffer containing the intracellular staining antibody cocktail and incubated for 30 min at room temperature. Additional wash steps were performed as described above for ICS.

### SARS-CoV-2 virus production

*C. aethiops* kidney cells (Vero E6; ATCC, CRL-1586) were maintained in high-glucose DMEM (Gibco, catalog no. 11965092) supplemented with 10% Serum Plus II FBS substitute (Millipore Sigma, catalog no. 14009C) and GlutaMAX (Thermo Fisher Scientific, catalog no. 35050061) at 37°C with 5% CO_2_.

SARS-CoV-2 USA-WA1/2020 (Washington strain; catalog no. NR-52281, BEI Resources, Manassas, VA). Virus was propagated in-house in Vero E6 cells. The internal lot used for this study was T75-12, with a stock concentration of 5.59 × 10^5^ PFU/ml (determined with Avicel plaque assay). Virus propagation used the above media with the following deviation: No Serum Plus II FBS substitute was used during the first 90-min period, and following uptake cells were maintained with 2% Serum Plus II FBS Substitute.

### SARS-CoV-2 challenge

Animals were identified with individually numbered ear tags before challenge. On study day 1, each animal was lightly sedated with isoflurane and received 30 μl of undiluted challenge virus intranasally. Clinical observations were performed daily to evaluate moribundity and weight loss from the day of challenge until study termination. A baseline body weight was recorded for each animal before challenge, and animals were weighed daily until euthanasia. Euthanasia was scheduled for all animals on study day 15 (14 dpi). A subset of mice was euthanized on study day 5 (4 dpi). Lung, nasal turbinate, and brain were collected for viral titer assessment (all tissues) and histology (lung only).

#### Time-weighted average calculation

The time-weighted average for either weight or clinical score is calculated as follows

$$\text{Time}\;-\;\text{weighted average}\;=\;\frac{\text{Area under the curve}\;\left(\text{AUC}\right)}{\text{number of days observed}}$$

The AUC and number of days observed are obtained from weight change percentage versus time or clinical score versus time plots for individual animals.

### Plaque assay

Up to 100 mg was cut from each harvested lung. This was then transferred to a 2-ml tube and homogenized to a total volume of 1 ml of DMEM (supplemented with 2% FBS) using a 5-mm stainless steel bead and a Tissuelyser II. Homogenization was carried out at 30 Hz for 2 min, repeated twice. The homogenized tissue was clarified by centrifugation, and the lung viral titer was quantified using crystal violet plaque assay.

The day before the assay, 8.0 × 10^5^ VeroE6 cells were seeded per well of a six-well plate in 2 ml of media. Plaque assays were carried out by first diluting the homogenized lung tissue in DMEM supplemented with 1× GlutaMAX, 1 mM sodium pyruvate, 10% FBS, and 1× nonessential amino acids (Gibco), and 200 μl of each dilution was added to the confluent monolayers of NR-596 Vero E6 cells (ATCC, #C1008) in duplicate and incubated in a 5% CO_2_ incubator at 37°C for 1 hour. The cells were rocked gently every 10 to 15 min to prevent monolayer drying. Cells were then overlaid with a 1:1 solution of 2.5% (v/v) Avicel RC-591 microcrystalline cellulose and carboxymethylcellulose sodium (DuPont Nutrition & Biosciences) and 2× modified Eagle’s medium (Temin’s modification, Thermo Fisher Scientific, catalog no. 10370088) supplemented with 100× antibiotic-antimycotic (Thermo Fisher Scientific, catalog no. 15240062) and 100× GlutaMAX both to a final concentration of 2× and 10% (v/v) FBS. The plates were then incubated at 37°C for 2 days (WA/2020 strain). After 2 days, the monolayers were fixed with 10% (v/v) neutral buffered formalin (NBF) (Thermo Fisher Scientific, catalog no. LC146705) for at least 6 hours and stained with 0.2% (v/v) aqueous Gentian Violet (Fisher Sci, catalog no. 3233-16) in 10% (v/v) NBF for 30 min, followed by rinsing and plaque counting.

### Statistical analysis

All statistical analyses were performed using GraphPad Prism 10. Tests used are specified in figure legends. Unless otherwise stated, *P < 0.05, **P < 0.01, ***P < 0.001, and ****P < 0.0001.
