# Supplementary material for: Conserved CD8 T cell vaccines without B cell epitopes drive robust protection against SARS-CoV-2 that is enhanced by intranasal boost
Source: Sci Adv. 2025 Nov 21;11(47):eadx0037. doi: 10.1126/sciadv.adx0037 (PMC12637294; doi:10.1126/sciadv.adx0037)
Supplement: Supplementary file 1 — Figs. S1 to S11 Tables S1 to S3 [file sciadv.adx0037_sm.pdf]

Science Advances
AAAS

Supplementary Materials for

Conserved CD8 T cell vaccines without B cell epitopes drive robust protection against SARS-CoV-2 that is enhanced by intranasal boost

Genghao Chen *et al.*

Corresponding author: Stephen J. Elledge, selledge@genetics.med.harvard.edu

Sci. Adv. **11**, eadx0037 (2025)
DOI: 10.1126/sciadv.adx0037

This PDF file includes:

Figs. S1 to S11
Tables S1 to S3

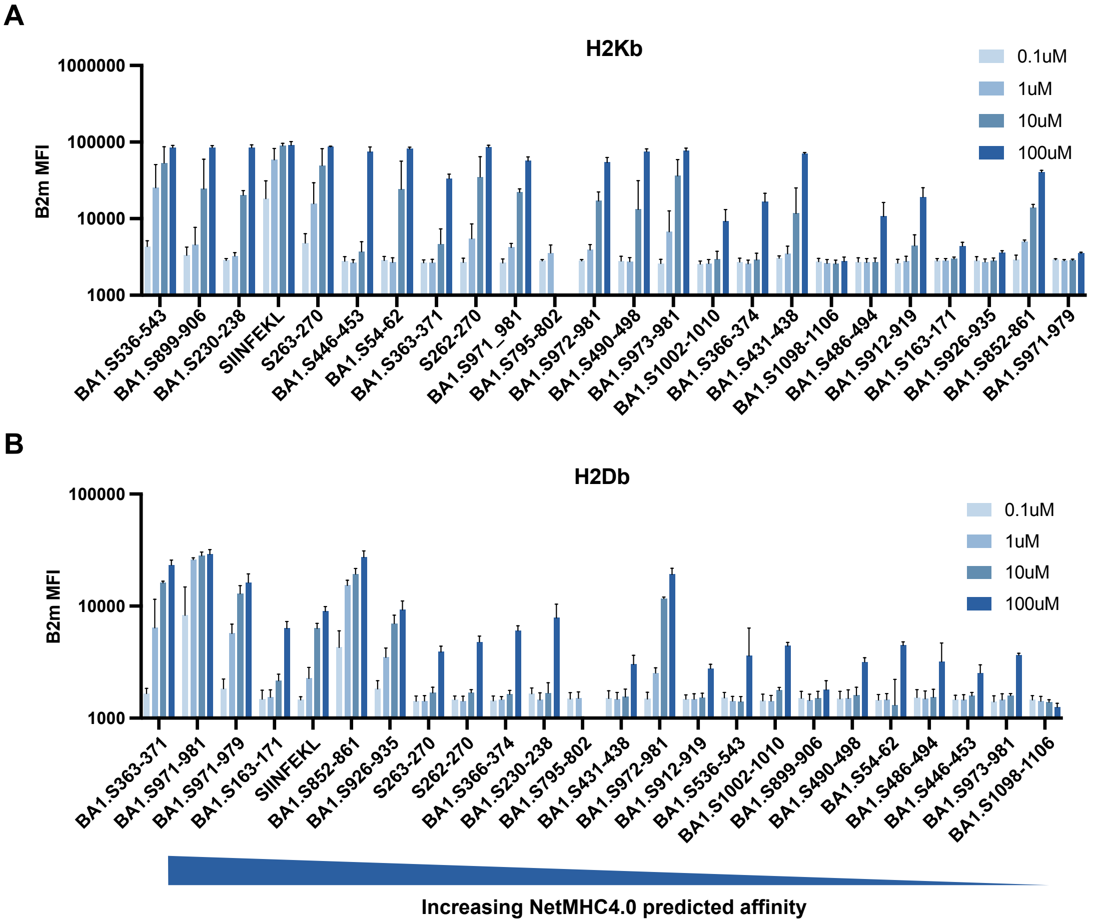

Figure S1. **Measurement of peptide-MHC binding stability for SARS-CoV-2 CD8 T cell epitopes.** MHC class I-, TAP1/TAP2- and ERAP1/ERAP2-null HEK293T (EpiScan) cells expressing either H-2Kb (**A**) or H-2Db (**B**) were pulsed with 0.1-100μM peptide. Cell surface stabilization of MHC-I was measured by β2m staining. N=2.

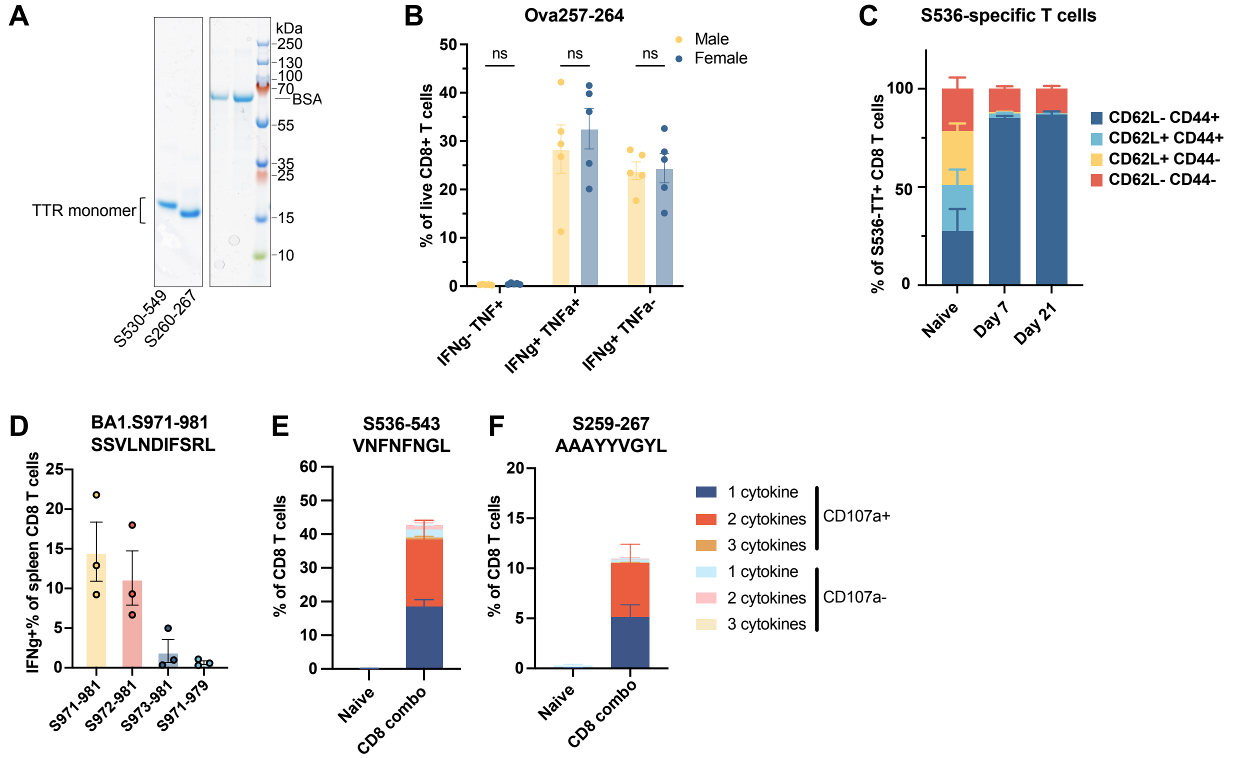

Figure S2. Characterization of CD8 T cell responses induced by TTR-epitope fusion vaccines. (A) SDS-PAGE of purified TTR-epitope fusion proteins visualized by Coomassie staining. BSA were used as reference. (**B**) T cell response in male and female mice following TTR immunizations of SIINFEKL. ICS was done to assess antigen-specific production of IFNg and TNFa by blood CD8 T cells. Mean ± SEM. N=5. (**C**) CD62L and CD44 expression of S536-specific T cells on day 7 (after prime) and on day 21 (after boost). Mean ± SEM. N=5. (**D**) ICS of splenocytes after 3 doses of TTR BA1.S971-981 to determine the optimal epitope. Splenocytes were collected 6 dafter the last immunization and re-stimulated with the indicated peptides on the x-axis. (**E-F**) Mice were vaccinated on d0 and d14 with CD8 combo (TTR-S536+TTR-S259) and ICS of blood CD8 T cells for IFNg, TNFa, IL-2 and CD107a was performed on d21. Frequencies of cells that secrete 1, 2 or 3 cytokines +/- CD107a expression are shown. (E) Cells stimulated with S536-543 peptide. (F) Cells stimulated with S259-267 peptide.

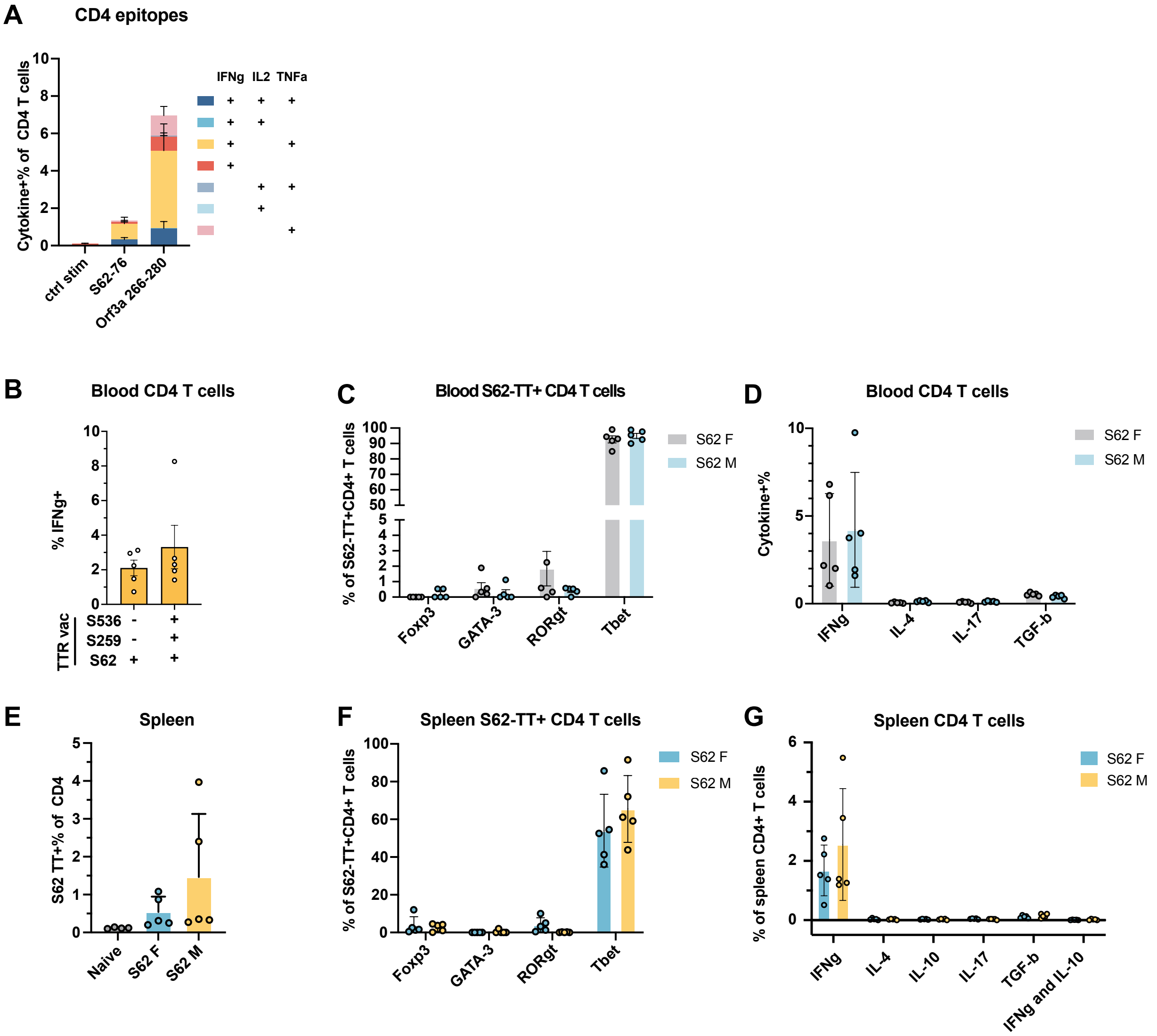

Figure S3. Characterization of CD4 T cell responses induced by TTR-epitope fusion vaccines. (**A**) ICS of blood CD4 T cells after immunization with TTR-S62-76 and TTR-Orf3a 266-280. Colors represent the combination of cytokine expressed. (**B**) CD4 T cell response to S62-76 after immunization of female C57BL/6 mice with TTR-S62-76 alone or in combination with CD8 epitopes (S536 and S259). Measured by ICS of blood on day 21. Mean± SEM. N=5. (**C-D**) Mice were primed on day 0, boosted on day 31 with TTR-S62-76 and bled on day 37 for (C) tetramer staining and TF expression analysis and (D) ICS. N=5. (**E-G**) T cell response was determined in the spleens of the same mice on day 51. N=4 for naïve, n=5 for all other groups. (E) Tetramer staining, mean ± SD. (F) TF expression in S62-tetramer^{+} CD4 T cells, mean ± SD. (G) ICS analysis of spleen CD4 T cells following overnight stimulation with S62-76 peptide. Mean ± SD.

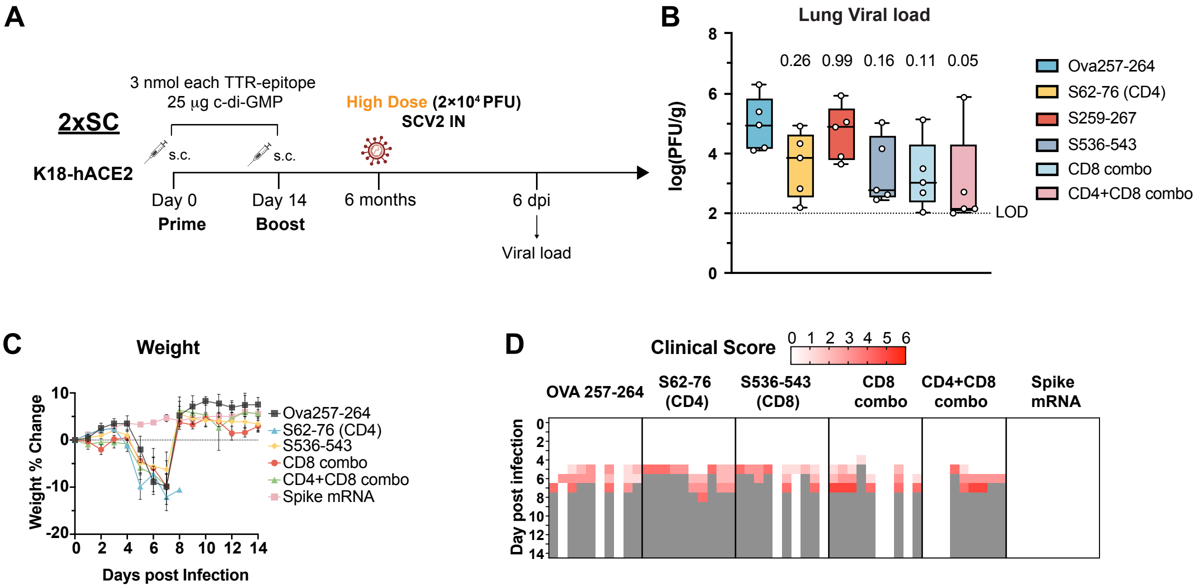

Figure S4. Subcutaneous immunizations of T cell epitopes reduce lung viral load in long term but failed to improve clinical outcomes of SARS-CoV-2 challenge. Related to Figure 3A-C. (**A**) Experimental scheme. Female K18-hACE2 mice were immunized with 3 nmol each TTR-epitope protein and 25 mg c-di-GMP subcutaneously and challenged 6 months later with 2×10^4 PFU (high-dose) 2019n-CoV/USA WA1/2020. On 6 dpi, mice were sacrificed, and lung viral load was determined by plaque assay. CD8 combo, contains S536-543 and S259-267. CD4+CD8 combo, contains S62-76, S536-543 and S259-267. N=5. (**B**) Box plot of lung viral load determined by plaque assay. Y-axis shows plaque-forming units (PFU) per gram of lung tissue in a log scale. One-way ANOVA and Tukey's test was performed and the adjusted p-value comparison to the Ova257-264 group is shown on the top. (**C-D**) Female K18-hACE2 mice were immunized with 3 nmol each TTR-epitope protein and 25 µg c-di-GMP subcutaneously and challenged with 2×10^4 PFU (high-dose) 2019n-CoV/USA WA1/2020 on indicated days. Mice were monitored for clinical symptoms and weighed for 14 d post infection (dpi). CD8 combo, contains S536-543 and S259-267. CD4+CD8 combo, contains S62-76, S536-543 and S259-267. Intramuscular Spike mRNA-LNP was used as a positive control, given at a dose of 1 µg. A cohort of mice were sacrificed on 6 dpi to measure lung viral load. (**C**) Weight change. Mean ± SEM is shown. N=10. (**D**) Clinical scores.

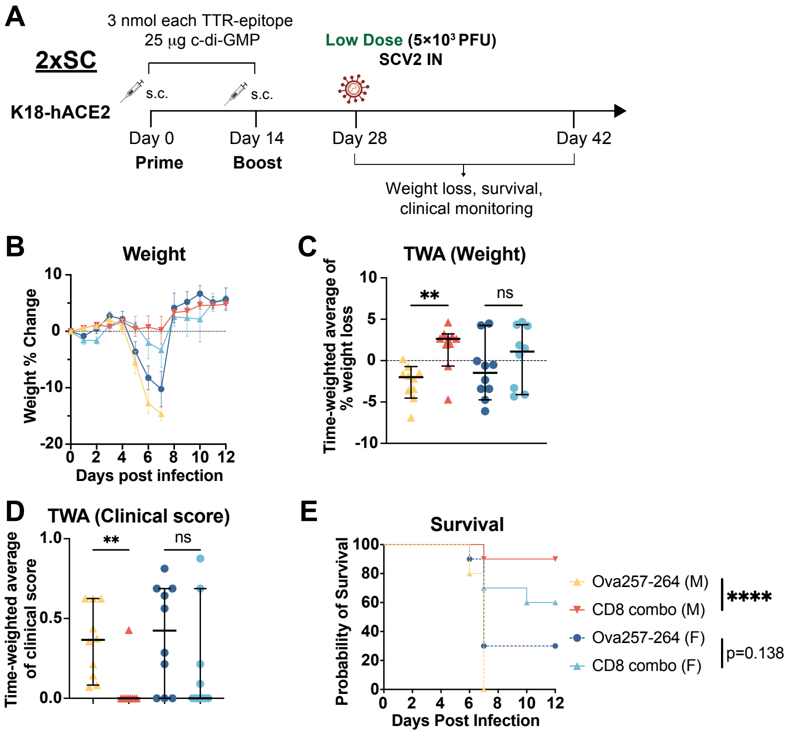

Figure S5. Subcutaneous immunization with SARS-CoV-2 CD8 T cell epitopes conferred protection against low-dose SARS-CoV-2 challenge in both male and female mice. Related to Figure 3D-I. (**A**) Experimental scheme. N=20 (10M, 10F). (**B**) Weight change. Mean ± SEM is shown. (**C**) Time-weighted average (TWA) of weight change calculated between 1-12 dpi. Median with 95%CI is shown. Kruskal-Wallis test with Dunn's correction was performed. (**D**) Time-weighted average of clinical scores shown in Figure 3H. Median with 95%CI is shown. Kruskal-Wallis test with Dunn's correction was performed. (**E**) Survival. log-rank Mantel-Cox test was performed.

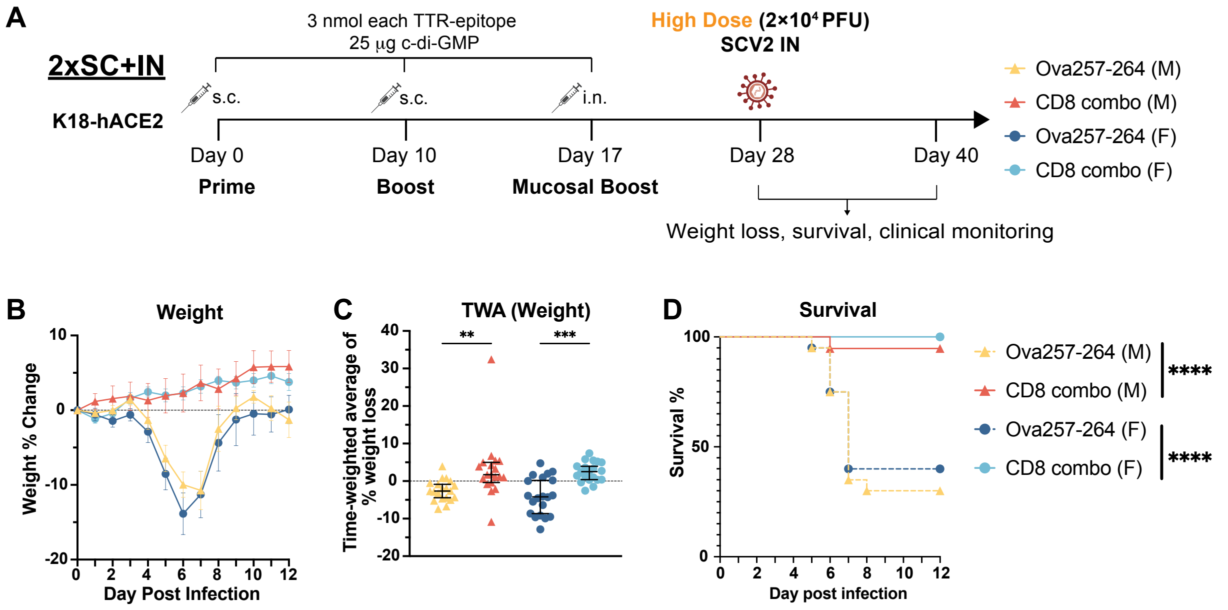

Figure S6. Intranasal boost of CD8 T cell epitopes provides protection against high-dose SARS-CoV-2 challenge in both male and female mice. Related to Figure 4F-J. (**A**) Experimental scheme. N=39 for CD8 combo (19M, 20F). N=40 for Ova257-264 (20M, 20F) (**B-C**) Weight change. (B) Weight change. Mean ± SEM is shown. (C) Time-weighted average of weight change calculated between 1-12 dpi. Median with 95%CI is shown. Kruskal-Wallis test with Dunn’s correction was performed. (**D**) Survival. log-rank Mantel-Cox test.

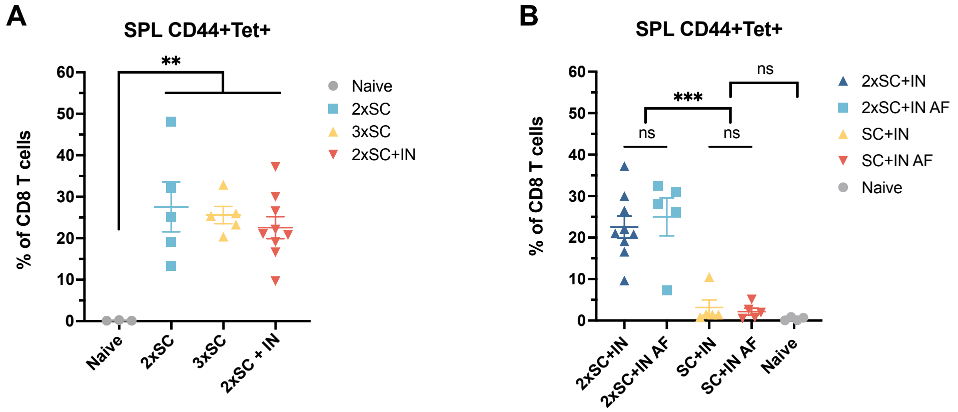

Figure S7. Splenic T cell responses following subcutaneous vaccinations or intranasal boosters. (**A**) Percentage of $CD44^{+}$ S536-$Tetramer^{+}$ cells in spleen CD8 T cells after different immunization schemes indicated in **Figure 4A**. One-way ANOVA and Tukey's test was used for comparisons. (**B**) Percentage of $CD44^{+}$ S536-$Tetramer^{+}$ cells in spleen CD8 T cells after different immunization schemes indicated in **Figure 5A**. One-way ANOVA and Tukey's test was used for comparisons.

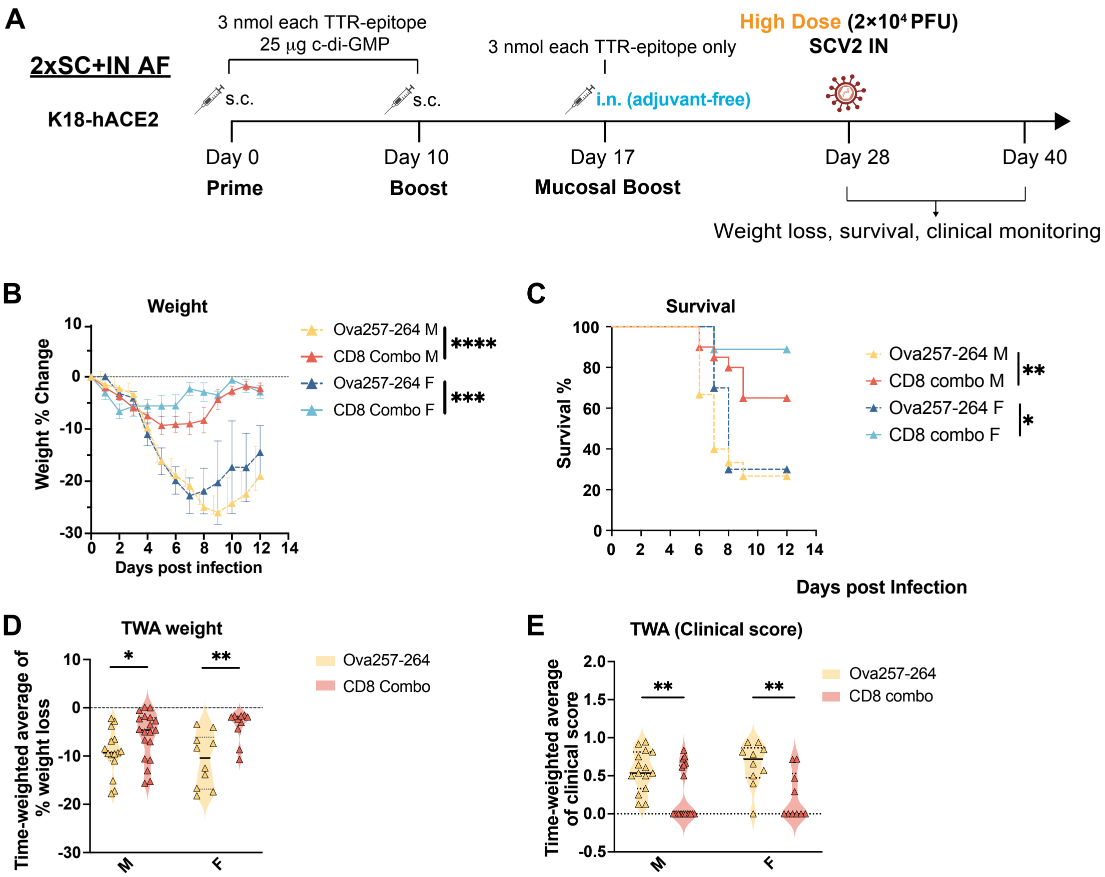

Figure S8. Adjuvant-free intranasal boost provides protection against high-dose SARS-CoV-2 challenge in both male and female mice. Related to Figure 5E-J. (**A**) Experimental scheme. N=25 for Ova257-264 (15M, 10F). N=29 for CD8 Combo (19M, 10F). (**B**) Weight change. Mean ± SEM is shown. A linear mixed-effects model was applied, where treatment and time were the two variables. P<0.0001 between treatment groups. (**C**) Survival. log-rank Mantel-Cox test. (**D**) Time-weighted average of weight change calculated between 1-12 dpi. Median with 95%CI. Kruskal-Wallis test with Dunn's correction. (**E**) Time-weighted average of clinical scores. Median with 95%CI is shown. Kruskal-Wallis test with Dunn's correction was performed.

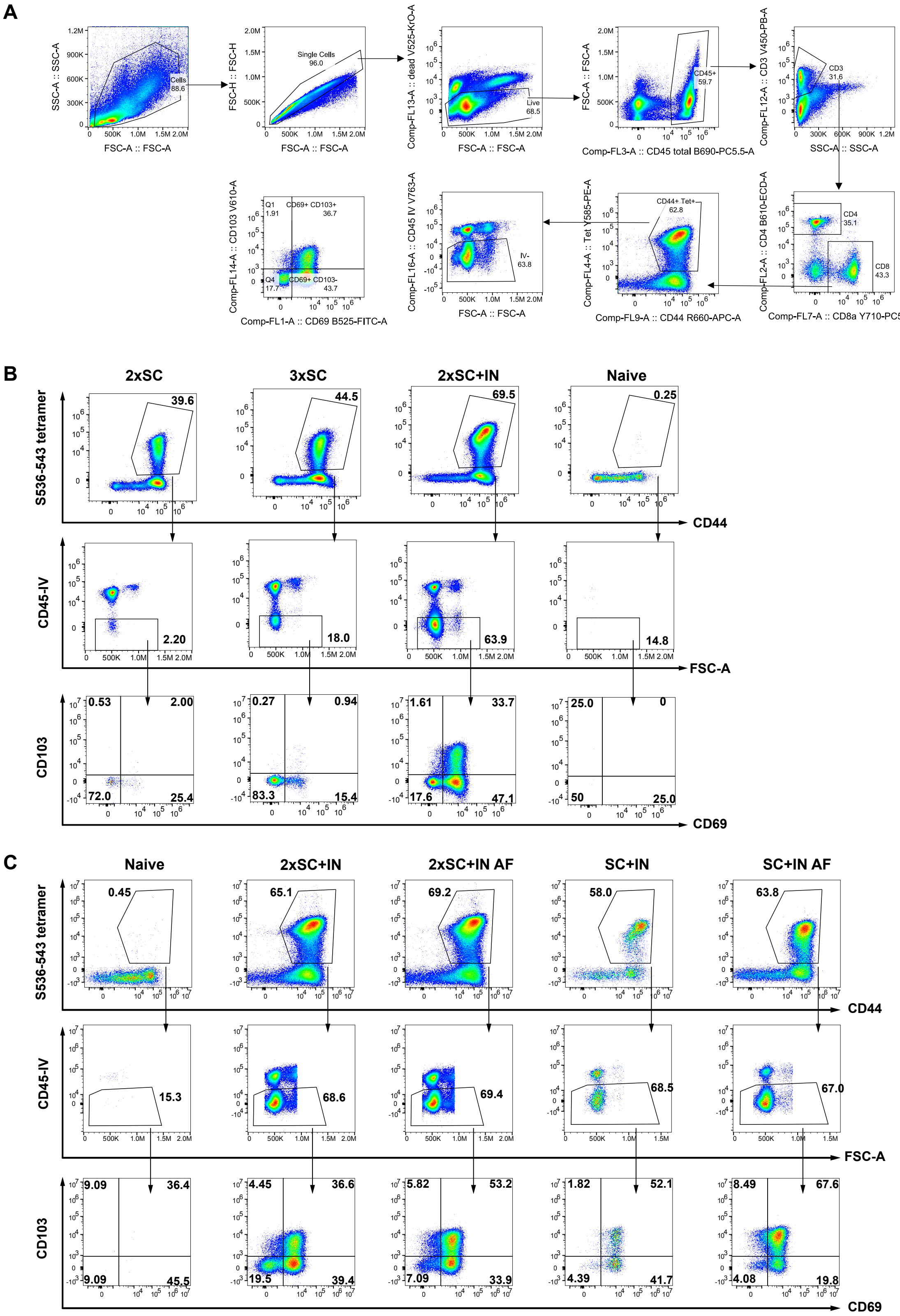

Figure S9. Gating strategy to identify T_{RM} cells and representative FACS plots for Figure 4B-D and Figure 5B-D. (A) Gating strategy to identify T_{RM} cells. (B) Representative FACS plots for Figure 4B-D. (C) Representative FACS plots for Figure 5B-D.

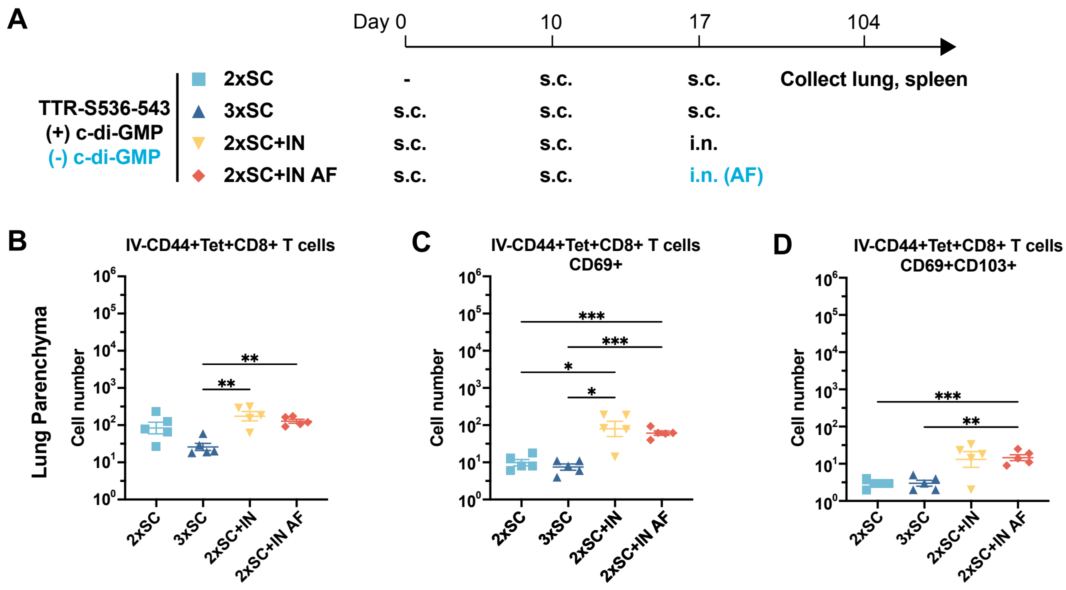

Figure S10. Adjuvant-free intranasal boost elicits lung T_{RM} responses that persisted long-term. Related to Figure 5. (**A**) Immunization scheme. Female C57BL/6 mice were immunized with 3 nmol TTR S536-543 and 25 µg c-di-GMP either subcutaneously (s.c.) or intranasally (i.n.) on indicated dates. Indicated intranasal immunizations (blue) were done without c-di-GMP. N=5 all groups. (**B**-**D**) S536-specific T cell response in the lung parenchyma on day 104 is quantified as (B) $CD45IV^-$ $CD44^+$ S536-tetramer$^+$, (C) $CD69^+$ $CD45IV^-$ $CD44^+$ S536-tetramer$^+$, and (D) $CD69^+$ $CD103^+$ $CD45IV^-$ $CD44^+$ S536-tetramer$^+$ $CD8^+$ T cells. Cell numbers per 10^5 total lung cells were shown.

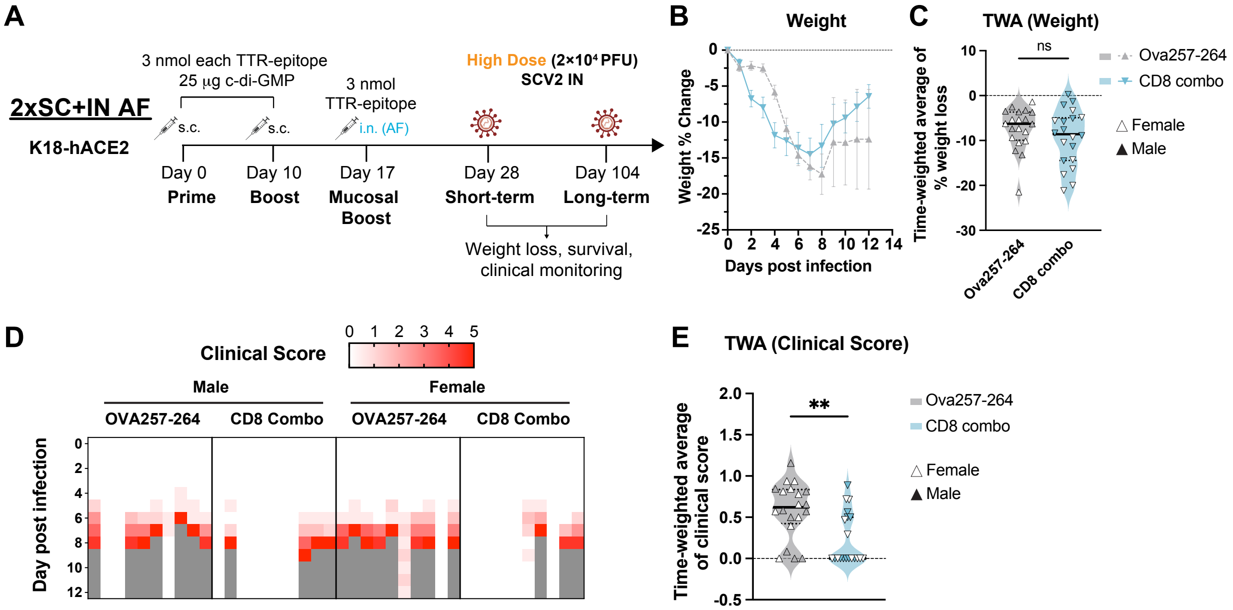

Figure S11. 2×SC+IN AF-administered T cell vaccines provided long-term protection against SARS-CoV-2. Related to Figure 5. (**A**) Experimental scheme for long-term protection study (same as Figure 5E). (**B-E**) Data from long-term protection study (day 104). (B) Weight change shown as mean ± SEM. (C) Violin plot shows time-weighted average of weight change calculated between 1-12 dpi. Filled and open symbols represent males and females, respectively. Median and quartiles are shown. Mann Whitney test was performed. (D) Clinical scores. Grey box indicates the animal was found dead or euthanized due to humanitarian endpoints. (E) Violin plot shows time-weighted average of clinical scores. Filled and open symbols represent males and females, respectively. Median and quartiles are shown. Mann Whitney test was performed.

Table S1. EC50 values of peptides in MHC-I stabilization assay.

Peptide	Sequence	EC50 (H-2K^b) (μM)	EC50 (H-2D^b) (μM)
S536-543*	VNFNFNGL	6.4	>100
S230-238*	INITRFQTL	65	>100
S259-267*	AAAYYVGYL	20	>100
S486-494*	YFPLRSYSF	>100	>100
S971-979*	SSVLNDIFS	>100	2.8
S490-498*	RSYSFRPTY	>100	>100
S926-935*	SAIGKIQDSL	>100	4.3
S973-981	VLNDIFSRL	15.0	>100
S446-453*	YNYLYRLF	>100	>100
S54-62*	LFLPFFSNV	39	>100
S852-861*	FKGLTVLPPL	37	0.88
S972-981	SVLNDIFSRL	40	8.8
S1002-1010*	QTYVTQQLI	>100	>100
S971-981*	SSVLNDIFSRL	65	0.05
S366-374*	YNLAPFFTF	>100	>100
S1098-1106*	HWFVTQRNF	>100	36
S363-371*	SVLYNLAPF	>100	4.9
Ova257-264	SIINFEKL	0.64	6.4

Table S1. Related to Figure S1. Residue position was determined in reference to Omicron BA.1 spike. EC50 was determined by fitting data to a three-parameter dose-response curve in GraphPad Prism 10. *Putative epitopes.

Table S2. TTR-fusion antigen sequences

Name	Sequence (Epitope underlined)
IgK Signal Peptide (used for all constructs except Ova257-264)	MRVPAQLLGLLLLWLPGARCA
Human Azurocidin preproprotein Signal Peptide (used for TTR Ova257-264)	MTRLTVLALLAGLLASSRA
TTR S62-76	GPAGAGESKCPLMVKVLDAVRGSPAVDVAVKVFKKTSEGSWEP<br>FASGKTAESGELHGLTTDEKFVEGVYRVELDTKSYWKTLGISPF<br>HEFADVVFTANDSGHRHYTIAALLSPYSYSTTAVVSNPQNGGGS<br>HHHHHHGGGSVTWFHAIHVSGTNGT
TTR Ova257-264	GPAGAGESKCPLMVKVLDAVRGSPAVDVAVKVFKKTSEGSWEP<br>FASGKTAESGELHGLTTDEKFVEGVYRVELDTKSYWKTLGISPF<br>HEFADVVFTANDSGHRHYTIAALLSPYSYSTTAVVSNPQNLLVY<br>SIINFEKLGGGSTGHHHHHH
TTR BA1.S536-543	GPAGAGESKCPLMVKVLDAVRGSPAVDVAVKVFKKTSEGSWEP<br>FASGKTAESGELHGLTTDEKFVEGVYRVELDTKSYWKTLGISPF<br>HEFADVVFTANDSGHRHYTIAALLSPYSYSTTAVVSNPQNGGGS<br>HHHHHHGGGSLVKNKCVNFNFNGLKGTGVL
TTR BA1.S259-267	GPAGAGESKCPLMVKVLDAVRGSPAVDVAVKVFKKTSEGSWEP<br>FASGKTAESGELHGLTTDEKFVEGVYRVELDTKSYWKTLGISPF<br>HEFADVVFTANDSGHRHYTIAALLSPYSYSTTAVVSNPQNGGGS<br>HHHHHHGGGSGWTAGAAAYYVGYL
TTR BA1.S971-981	GPAGAGESKCPLMVKVLDAVRGSPAVDVAVKVFKKTSEGSWEP<br>FASGKTAESGELHGLTTDEKFVEGVYRVELDTKSYWKTLGISPF<br>HEFADVVFTANDSGHRHYTIAALLSPYSYSTTAVVSNPQNGGGS<br>HHHHHHGGGSSKFGAISSVLNDIFSRLDKV
TTR ORF3a 266-280	GPAGAGESKCPLMVKVLDAVRGSPAVDVAVKVFKKTSEGSWEP<br>FASGKTAESGELHGLTTDEKFVEGVYRVELDTKSYWKTLGISPF<br>HEFADVVFTANDSGHRHYTIAALLSPYSYSTTAVVSNPQNGGGS<br>HHHHHHGGGSEPIYDEPTTTTSVPL

Table S3. List of flow cytometry reagents.

Reagent	Fluorophore	Clone	Source
anti-mouse CD3	PerCP-efluor710	17A2	Thermo 46-0032-82
anti-mouse CD4	RB613	RM4-5	BD 571284
anti-mouse CD8a	RY703	53-6.7	BD 571447
anti-mouse CD44	APC	IM7	Biolegend 103011
anti-mouse CD69	FITC	H1.2F3	Biolegend 104505
anti-mouse CD103	BV605	2E7	Biolegend 121433
anti-mouse/human CD11b	APC/Fire750	M1/70	Biolegend 101261
anti-mouse F4/80	APC/Fire750	BM8	Biolegend 123151
anti-mouse/human CD45R/B220	APC/Fire750	RA3-6B2	Biolegend 103259
anti-mouse CD11c	APC/Fire750	N418	Biolegend 117351
anti-mouse CD45	BV421	30-F11	Biolegend 103133
anti-mouse CD45	BV785	30-F11	Biolegend 103149
Zombie Aqua Fixable Viability			Biolegend 423101
anti-mouse CD8a	BV605	53-6.7	Biolegend 100743
anti-mouse CD3	BV421	17A2	Biolegend 100227
anti-mouse IFNg	APC	XMG1.2	Biolegend 505809

anti-mouse TNFa	PE	MP6-XT22	Biolegend 506305
S536-543 H-2K(b) Tetramer	PE		NIH Tetramer Core facility
anti-mouse IL-2	FITC	JES6-5H4	Biolegend 503806
anti-human β2-microglobulin	APC	A17082A	Biolegend 154506
anti-mouse IL-4	Alexa Fluor 488	11B11	Biolegend 504111
anti-mouse IL-17A	BV605	TC11-18H10.1	Biolegend 506927
anti-mouse LAP (TGF-β1)	BV421	TW7-16B4	Biolegend 141407
anti-GATA3	BV421	16E10A23	Biolegend 653813
anti-mouse RORγt	RB705	Q31-378	BD 570260
anti-T-bet	RB613	4B10	BD 571346
anti-FoxP3	PE	FJK-16s	Thermo 12-5773-80
anti-mouse IL-10	PE/Cyanine7	JES5-16E3	Biolegend 505025
anti-mouse CD62L	FITC	MEL-14	Biolegend 104406
